# Phenotypic heterogeneity promotes adaptive evolution

**DOI:** 10.1371/journal.pbio.2000644

**Published:** 2017-05-09

**Authors:** Zoltán Bódi, Zoltán Farkas, Dmitry Nevozhay, Dorottya Kalapis, Viktória Lázár, Bálint Csörgő, Ákos Nyerges, Béla Szamecz, Gergely Fekete, Balázs Papp, Hugo Araújo, José L. Oliveira, Gabriela Moura, Manuel A. S. Santos, Tamás Székely Jr, Gábor Balázsi, Csaba Pál

**Affiliations:** 1 Synthetic and Systems Biology Unit, Biological Research Centre, Szeged, Hungary; 2 Department of Systems Biology - Unit 950, The University of Texas MD Anderson Cancer Center, Houston, Texas, United States of America; 3 School of Biomedicine, Far Eastern Federal University, Vladivostok, Russia; 4 DETI & IEETA, University of Aveiro, Aveiro, Portugal; 5 Department of Medical Sciences and Institute of Biomedicine - iBiMED, University of Aveiro, Aveiro, Portugal; 6 The Louis and Beatrice Laufer Center for Physical and Quantitative Biology, Stony Brook University, Stony Brook, New York, United States of America; 7 Department of Biomedical Engineering, Stony Brook University, Stony Brook, New York, United States of America; New York University, United States of America

## Abstract

Genetically identical cells frequently display substantial heterogeneity in gene expression, cellular morphology and physiology. It has been suggested that by rapidly generating a subpopulation with novel phenotypic traits, phenotypic heterogeneity (or plasticity) accelerates the rate of adaptive evolution in populations facing extreme environmental challenges. This issue is important as cell-to-cell phenotypic heterogeneity may initiate key steps in microbial evolution of drug resistance and cancer progression. Here, we study how stochastic transitions between cellular states influence evolutionary adaptation to a stressful environment in yeast *Saccharomyces cerevisiae*. We developed inducible synthetic gene circuits that generate varying degrees of expression stochasticity of an antifungal resistance gene. We initiated laboratory evolutionary experiments with genotypes carrying different versions of the genetic circuit by exposing the corresponding populations to gradually increasing antifungal stress. Phenotypic heterogeneity altered the evolutionary dynamics by transforming the adaptive landscape that relates genotype to fitness. Specifically, it enhanced the adaptive value of beneficial mutations through synergism between cell-to-cell variability and genetic variation. Our work demonstrates that phenotypic heterogeneity is an evolving trait when populations face a chronic selection pressure. It shapes evolutionary trajectories at the genomic level and facilitates evolutionary rescue from a deteriorating environmental stress.

## Author summary

Phenotypic heterogeneity of genetically identical cells can generate nonheritable variation in a population. Is this heterogeneity favorable for microbes? In a changing environment, the answer is a definite yes. While scholars have argued that stochastically generated variation precedes genetic changes and thereby facilitate the evolution of complex traits, this idea has remained disputed, not least because of the shortage of experimental studies. We address this long-standing and controversial issue by integrating synthetic biology, laboratory experimental evolution, and genomic analyses. We explicitly tested the mechanisms whereby phenotypic heterogeneity may promote evolvability. Our work demonstrates that phenotypic heterogeneity facilitates evolutionary rescue from deteriorating environmental stress by generating individuals with exceptionally high fitness. Remarkably, elevated phenotypic heterogeneity evolves as a direct response to stress and thereby it promotes evolution of rare combinations of mutations. These results indicate that phenotypic heterogeneity might have an important role in the evolution of key innovations.

## Introduction

According to classical evolutionary theory, genetic variation provides the major source of heritable variation on which natural selection acts. However, many scholars argue that phenotypic heterogeneity can accelerate adaptive evolution [1–3]. Phenotypic heterogeneity can take many forms, including stochastic gene expression variability [4], alternative protein conformations [5], morphological plasticity [3], cellular age-correlated phenotypic plasticity [6], and learning throughout the lifetime of the organism [7,8]. The literature goes back to Baldwin’s suggestion in 1896 [7] and has recently been discussed extensively [3,4,9,10]. Phenotypic heterogeneity has been shown to have a beneficial effect in fluctuating environments [11–14]. However, its role in long-term adaptation has remained disputed [10,15,16], partly due to the shortage of experimental evolutionary studies (but see [17]). This issue is important, not least because phenotypic heterogeneity may facilitate evolution of microbial drug resistance [18] and cancer progression [19]. One prior study claims that the direction of phenotypic plasticity in gene expression is generally opposite to the direction of adaptive evolution [20] (see also [15]).

Based on prior theoretical work [21], we hypothesized that phenotypic heterogeneity has a long-lasting impact on adaptive evolution for two possible reasons. Phenotypic heterogeneity may increase population size and hence the chance of occurrence of adaptive mutations. Alternatively, by generating individuals with exceptionally high trait values, phenotypic heterogeneity may increase the net adaptive value of beneficial mutations at an early stage of adaptation.

In this work, we study phenotypic heterogeneity that arises from stochastic fluctuation in cellular states and focus on the impact of such nongenetic cellular variation under permanent challenges in a novel stressful environment. Specifically, we developed two versions of an inducible synthetic gene circuit that generate varying degrees of expression stochasticity of an antifungal resistance gene. We tested the costs and benefits of phenotypic heterogeneity under stress and nonstress conditions alike. Next, we investigated how these two genetic circuits influence evolutionary adaptation towards antifungal stress and what the underlying molecular mechanisms of adaptation might be. Specifically, we asked how the level of nongenetic cellular variation shapes mutational effects. We provide several lines of evidence that phenotypic heterogeneity promotes evolvability, partly by modulating the adaptive value of beneficial mutations.

## Results

### Genetic circuits conferring phenotypic heterogeneity

We first developed and characterized two versions of a chromosomally integrated, inducible synthetic gene circuit that generate varying degrees of expression stochasticity of an antifungal resistance gene. The positive feedback (PF) gene circuit consisted of a modified version of the reverse-tetR trans-activator coding gene (*rtTA-MF*) [22,23], which is under the control of a synthetic tet-inducible tetreg promoter (P_Tetreg2_). When bound by doxycycline (a tetracycline analog), the reverse-tetR trans-activator protein (rtTAp) activates the expression of its own gene as well as the C-terminally *GFP*-tagged *PDR5* target gene (*PDR5-GFP*; also controlled by P_Tetreg2_) [22,24]. Many similar positive regulatory feedback loops, leading to alternative heritable phenotypes, have been described in microbes [1].

**Fig 1:**
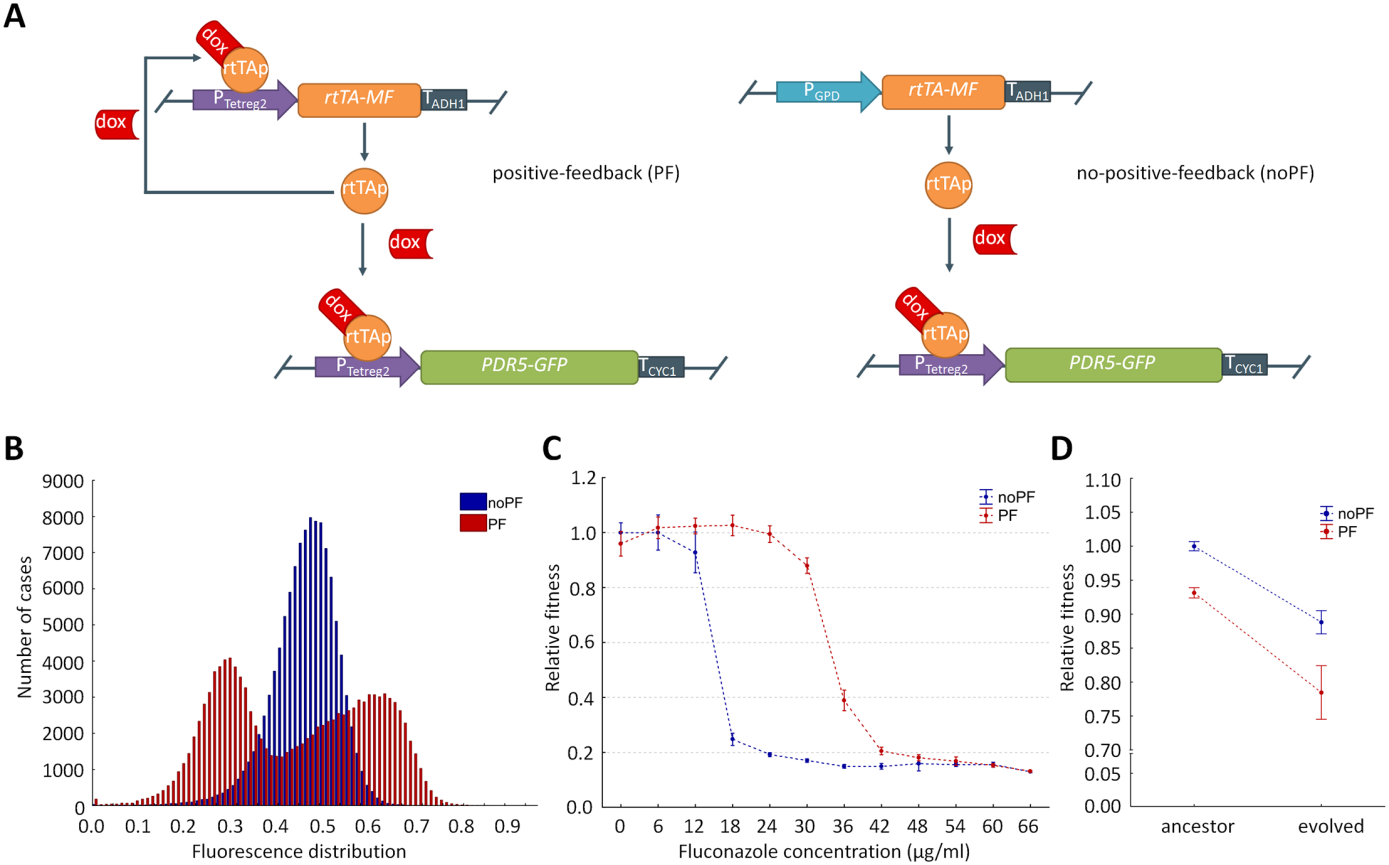
Basic properties of the synthetic genetic circuits. **(A) Elements of the synthetic constructs.** The synthetic constructs comprise the reverse-tetR trans-activator (*rtTA-MF*) gene and the C-terminally *GFP*-tagged *PDR5* reporter gene (*PDR5-GFP*). The *PDR5-GFP* reporter gene is controlled by a synthetic tet-inducible tetreg promoter (P_tetreg2_), activated by doxycycline (dox). Doxycycline forms a complex with the internally produced reverse-tetR transactivator protein (rtTAp). The complex activates the expression of the reporter gene through binding to the tetO2 sites of P_tetreg2_. The two circuits differ only in the promoter of the *rtTA-MF* gene. In the case of the positive feedback (PF) circuit, the promoters of the *rtTA-MF* and the *PDR5-GFP* reporter gene are the same (P_tetreg2_), generating a positive regulatory loop. The no positive feedback (noPF) circuit has no such loop, as the *rtTA-MF* is driven by a constitutive promoter (glyceraldehyde-3-phosphate dehydrogenase promoter [P_GPD_], glyceraldehyde-3-phosphate dehydrogenase [*TDH3*]). Transcription of the *rtTA-MF* gene and the *PDR5-GFP* reporter gene was terminated by using the terminator sequence of alcohol dehydrogenase 1 gene (t_*ADH1*_) and cytochrome c (t_*CYC1*_), respectively. **(B) Monitoring the fluorescence level of the reporter gene by flow cytometry.** Fluorescence histograms of C-terminally GFP-tagged Pdr5p protein (Pdr5p-GFP) for selected PF and noPF strains, respectively. The noPF population showed low gene expression stochasticity, with unimodal expression distribution, and did not contain cells with extremely low or extremely high fluorescence levels. The difference in the mean target gene expression level was only 5%, and it was actually higher in the noPF-carrying strain, while the coefficient of variation differed by 150% between PF and noPF populations (Mann–Whitney *U* Test, *p* < 0.001). Measured fluorescence intensity was normalized to the forward scatter values (both log_10_-scaled). The inducer levels were 0.015 μg/ml (noPF) and 0.3 μg/ml (PF), respectively. **(C) Evaluation of fluconazole minimum inhibitory concentration (MIC) values of noPF and PF strains.** The figure shows the mean relative fitness of the ancestor PF (red lines) and noPF strains (blue line) as a function of fluconazole dosage. Absolute fitness was estimated by the increment of the optical density at 600 nm (OD_600_) after 72 h of growth of each strain at each fluconazole concentration (see Materials and methods). Relative fitness was calculated by normalizing the absolute fitness of each strain at each concentration to the noPF absolute fitness value in drug-free medium (see Materials and methods for details). Error bars indicate 95% confidence interval, based on 32 independent cultures grown and measured for OD_600._ MIC is defined at a 0.15 cutoff value of the increment of the OD_600_. **(D) Fitness cost of phenotypic heterogeneity.** The figure shows the mean relative fitness of the ancestor and the evolved strains in fluconazole-free medium. Evolved strains from the final day of the laboratory evolution experiment (Experiment A) were used for the analysis. Prior to evolution (ancestor), PF fitness is 7% lower than noPF fitness (Mann–Whitney *U* test, *p* < 0.001). Laboratory evolved strains (evolved) show fitness deficits in drug free medium, compared to their corresponding ancestors (11% in noPF, Mann–Whitney *U* test, *p* < 0.001; 16% in PF strains, Mann–Whitney *U* test, *p* < 0.001). Absolute fitness was estimated by the increment of the OD_600_ after 72 h of growth in drug-free medium (see Materials and methods). Relative fitness was calculated by normalizing the absolute fitness of each strain to the absolute fitness of the noPF ancestor. Error bars indicate 95% confidence interval, based on at least 140 independent cultures grown and measured for OD_600._ The data underlying Fig 1 can be found in S1 Data.

The second gene circuit (no positive feedback [noPF]) has a constitutively expressed *rtTA-MF* (controlled by glyceraldehyde-3-phosphate dehydrogenase promoter [P_GPD_]), as it lacks the positive feedback loop (Fig 1A). In both gene circuits, the rtTAp controls the expression of the bifunctional C-terminally GFP-tagged Pdr5p protein (Pdr5p-GFP) in *S*. *cerevisiae*. Pdr5p is a natural multidrug transporter involved in the efflux of several major antifungal drugs [25,26]. This bifunctional protein served as a fluorescent reporter, while simultaneously protecting cells from the antifungal agent fluconazole. Therefore, we could relate gene expression stochasticity to the corresponding variation in fitness. The native *PDR5* gene was eliminated in the genomes of both strains. Evolution of resistance mechanisms in the laboratory is contingent upon *PDR5* expression [25].

Flow cytometry was used to establish the distribution of the steady-state expression level of the target protein across cells from isogenic yeast cell populations carrying the PF or the noPF gene circuits, respectively. For both genotypes, the mean and the coefficient of variation (CV) of *PDR5-GFP* expressions were calculated. In agreement with a prior study [22], genotypes carrying the PF gene circuit showed bimodal expression (Fig 1B). This pattern reflects stochastic transitions between two cellular states that form distinct subpopulations, and it arises from the positive regulatory loop along with the nonlinear promoter response [22]. By contrast, the noPF gene circuit displayed unimodal expression distribution (Fig 1B). By varying the level of inducer concentration (S1 Fig), we found a regime where gene expression variability (estimated by the CV of the distribution) of the PF circuit was 150% larger (Fig 1B). In contrast, the difference in the mean target gene expression level was only 5%, and it was actually higher in the noPF-carrying strain (Fig 1B). Therefore, any potential evolutionary advantage of the PF gene circuit cannot be attributed to differences in mean *PDR5-GFP* expression between the two strains. Accordingly, the only main difference between the PF and noPF strains was the CV of the *PDR5-GFP* expression, making it possible to directly test the physiological and evolutionary impacts of gene expression stochasticity. The PF and noPF strains were induced by 0.3 μg/ml and 0.015 μg/ml doxycycline, respectively. Unless otherwise indicated, these inducer concentrations were used throughout the study. The switching rate between the expression states is between 10^−1^–10^−3^ per hour [22].

### Physiological costs and benefits of phenotypic heterogeneity

The PF and noPF genetic circuits were appropriately induced, and the corresponding growth patterns were investigated. The PF genotype shows enlarged expression variability, and thereby, it generated individuals with exceptionally high and low *PDR5-GFP* expression. Individuals with high *PDR5-GFP* expression are better able to cope with fluconazole stress, leading to the survival of a small subset of the population carrying the PF circuit [22]. On the other hand, production of an excess level of Pdr5p-GFP is expected to be costly in the absence of stress. In agreement with expectation, when cells were exposed to fluconazole at low dosages, the genotype carrying the PF genetic circuit was better able to propagate (Fig 1C), presumably due to a subset of the cell population with exceptionally high Pdr5p-GFP level [27]. These results indicate that differences in the level of cell-to-cell variability have an impact on fitness under environmental stress [27]. In contrast, PF cells showed somewhat reduced fitness in stress-free medium (Fig 1D). Along with a prior paper [28], these results indicate a trade-off between gene expression costs and survival under stress conditions, which may shape phenotypic heterogeneity in nature.

### Phenotypic heterogeneity promotes genetic adaptation

**Fig 2:**
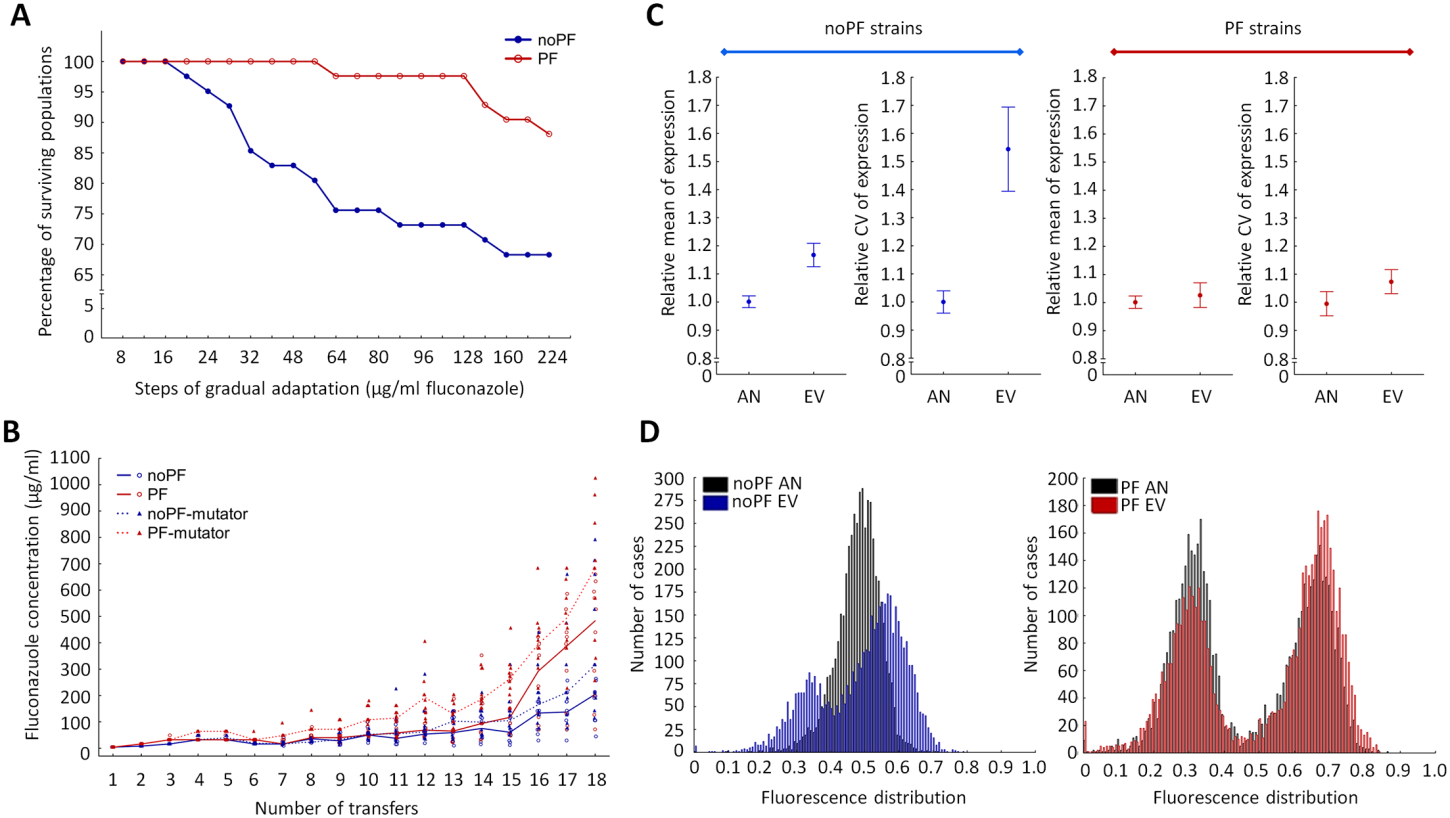
Impact of phenotypic heterogeneity on adaptive evolution. Two complementary experiments were used to study the impact of phenotypic heterogeneity on adaptive evolution. Experiment A measured extinction rate of the evolving strains as a function of gradually increasing fluconazole dosage (for further details see S2A Fig), while experiment B aimed to maximize the fluconazole resistance increment during a fixed time period (for further details, see S2B Fig). **(A) Outcome of Experiment A.** The figure shows the extinction dynamics of independently evolving strains exposed to gradually increasing fluconazole dosages. There was a significant difference in the ratio of surviving strains between no positive feedback (noPF) and positive feedback (PF) populations, respectively (paired *t*-test, *p* < 0.05). 32% of the noPF populations failed to adapt to the final employed fluconazole dosage (224 μg/ml), while the same figure was as low as 12% for PF (chi-squared test, *p* < 0.05). Evolved strains from the final day of Experiment A were used for further genomic and functional analyses. **(B) Outcome of Experiment B.** Ten independent populations of noPF (blue circles), PF (red circles), noPF mutator (blue triangle), and PF mutator (red triangle) strains were each allowed to evolve in parallel using an established automated evolution protocol (see Materials and methods). The figure shows the distribution of the level of resistance (i.e., the highest fluconazole dosage the strain could grow) as a function of time (transferring steps). The median line of the ten independent populations in each strain are color coded as follows: blue continuous line denotes noPF, red continuous line denotes PF, blue dashed line denotes noPF mutator, and red dashed line denotes PF mutator lines. At the end of Experiment B (i.e., at the last four transferring steps), there was a significant difference in the resistance level between PF nonmutator and noPF nonmutator strains (*t*-test, *p* < 0.05) and also between PF mutator and noPF mutator strains (*t*-test, *p* < 0.05), respectively. **(C) Properties of C-terminally *GFP*-tagged *PDR5* gene (*PDR5-GFP*) expression after evolution**. The figure shows the mean and coefficient of variation (CV) of *PDR5-GFP* expression distribution in the noPF and PF strains. The values were normalized to the corresponding ancestors, respectively. Mean expression level did not change significantly in the evolved PF strains (Mann–Whitney *U* test, *p* = not significant), while it increased by an average 17% in the noPF strains (Mann–Whitney *U* test, *p* < 0.001). The CV showed an average 54% increment in the evolved noPF strains (Mann–Whitney *U* test, *p* < 0.001), while the same increment was only 7% in the evolved PF strains (Mann–Whitney *U* test, *p* < 0.05). Error bars indicate 95% confidence interval. AN, ancestor; EV, evolved. **(D) C-terminally GFP-tagged Pdr5p protein (Pdr5p-GFP) fluorescence distribution after laboratory evolution.** The fluorescence histograms show the Pdr5p-GFP distribution of one representative laboratory evolved PF (red) and noPF (blue) strain, respectively, and the corresponding ancestors (black). For the characteristics of the distribution in all evolved strains, see S6 Fig. The data underlying Fig 2 can be found in S1 Data.

Does phenotypic heterogeneity influence genetic adaptation? We initiated laboratory evolutionary experiments with genotypes carrying either the PF or the noPF genetic circuits (Experiment A). Populations were cultivated in parallel (42 replicate populations per genotype): 10% of each culture was diluted into fresh medium every 72 h, and populations were allowed to evolve to progressively higher fluconazole stress for approximately 120 generations. Starting with a subinhibitory fluconazole concentration (8 μg/ml), the dosage was increased gradually (1.5 times the previous dosage) at every second transfer (S2A Fig). Several populations became extinct (i.e., showed no detectable growth after transfer), while others reached clinically significant levels of fluconazole resistance (up to 224 μg/ml) during the course of laboratory evolution (Fig 2A). Evolution of resistance caused an 11%–16% fitness deficit in drug-free medium (Fig 1D).

Prior work [25] suggests that stepwise increase of fluconazole concentrations promotes the evolution of resistance mechanisms, which are contingent upon *PDR5* expression. Therefore, stochastic expression variation in this gene is expected to influence evolutionary processes. This was indeed the case (Fig 2A): 32% of the noPF populations died out during the course of laboratory evolution, while the same figure was as low as 12% for PF populations. These results indicate that phenotypic heterogeneity had a major impact on extinction patterns in evolving populations facing fluconazole stress.

To confirm that phenotypic heterogeneity accelerates evolutionary adaptation, we initiated a new round of laboratory evolution with slightly modified experimental settings (Experiment B). Instead of measuring extinction rate, this protocol aimed to maximize the level of fluconazole resistance in the evolving population reached during a fixed time period (see Materials and methods, S2B Fig). Fig 2B shows the distribution of minimum inhibitory concentration (MIC) values in ten parallel evolved noPF and ten parallel evolved PF strains, respectively. We found that PF populations reached significantly higher fluconazole resistance during the course of laboratory evolution (Fig 2B and S3 Fig). This result does not simply reflect the initial difference in fluconazole susceptibilities between the PF and noPF genotypes, as the rate of adaptation (measured by relative increase in MIC levels) also differed: after only 110 generations, the PF populations reached an average 12-fold increase in MIC level relative to their ancestor, while the same figure was 8-fold in the case of noPF populations (Fig 2B).

### Mutational supply and phenotypic heterogeneity

Phenotypic heterogeneity may increase the rate of adaptation in two fundamentally different ways. It may elevate the rate by which beneficial mutations arise in the population (mutational supply theory). Indeed, the product of beneficial mutation rate and effective population size determines mutation supply rate in the population. A second potential mechanism to increase adaptation leaves population size and mutation rate unchanged but increases the beneficial effects of mutant alleles (mutational effect theory). Here, we investigate the first possibility.

One may argue that by increasing fitness, phenotypic heterogeneity increases population size and thereby the probability that a beneficial mutation will arise in the population. However, there was no significant difference in population size between evolving PF and noPF lineages (S4 Fig). Moreover, we failed to find evidence that the specific promoters underlying the PF genetic circuit would generate a local mutational hot spot (S1 Table), not least because the evolved PF strains did not accumulate an especially high number of mutations in the genetic circuit, in the Pdr5p coding region, or elsewhere in the genome (S2 Table). Similarly, there is no evidence that PF strains would have an especially high genomic mutation rate (S5 Fig). These results indicate that despite similar population densities and mutation rate, PF populations evolve more rapidly to fluconazole stress (Fig 2B).

We finally asked how elevated mutational supply affects the outcome of laboratory evolution. As controlling population size is cumbersome and can potentially alter selection pressure, we manipulated genomic mutation rate. Briefly, a mismatch-repair gene (*MSH2*) was inactivated in the PF and noPF strains, respectively, leading to an approximately 10-fold increase in genomic mutation rate, in accordance with previous works [29,30]. We initiated laboratory evolution with the mutator and nonmutator strains, as described previously (Experiment B, Fig 2B). As expected [31], mutator strains (*Δmsh2*) reached higher levels of fluconazole resistance than the corresponding nonmutators that carried the same genetic circuit (PF or noPF, respectively). More surprisingly, the level of resistance in the evolved noPF mutator strains was consistently lower than that in the evolved PF nonmutator strains (Fig 2B). This suggests that despite massive increase in mutational supply, the genotype with low phenotypic heterogeneity has an intrinsic disadvantage during evolutionary adaptation. We conclude that the observed low adaptation rate under low phenotypic heterogeneity cannot be explained by shortage of mutational supply only.

### De novo evolution of phenotypic heterogeneity

The above results demonstrated that phenotypic heterogeneity promotes adaptation rate under prolonged exposure to fluconazole stress. Under the assumption that evolution favors individuals with exceptionally high trait values, one might also expect evolution of elevated phenotypic heterogeneity as a secondary response. Specifically, the selection pressure towards broader Pdr5p-GFP distribution should be especially strong when the level of phenotypic heterogeneity is initially low, such as in noPF strains. Moreover, the mean *PDR5-GFP* expression should also increase. This was indeed so.

We isolated single clones from 27 independently evolved noPF and 27 independently evolved PF strains, respectively, each from the final day of the evolutionary experiments (Experiment A). The distribution of the target protein expression level was estimated in these strains and their corresponding ancestors. The CV showed an average 54% increment of the initial value in the evolved noPF strains, while only a very modest 7% change was found in the evolved PF strains (Fig 2C). Similar trends were observed for changes in the mean expression level: no significant changes were detected in the evolved PF strains, while the noPF strains showed an average 17% increment (Fig 2C). In the evolved noPF strains, the distribution of Pdr5p-GFP fluorescence deviated significantly from normal distribution (Fig 2D and S6B Fig), and, in at least some cases, it appeared to be bimodal (S6A Fig). These results indicate that under strong directional selection for exceptionally high Pdr5p-GFP level, both the mean and the breadth of expression evolve. For molecular underpinnings of these alterations, see S1 Table.

### Phenotypic heterogeneity shapes mutational effects

**Fig 3:**
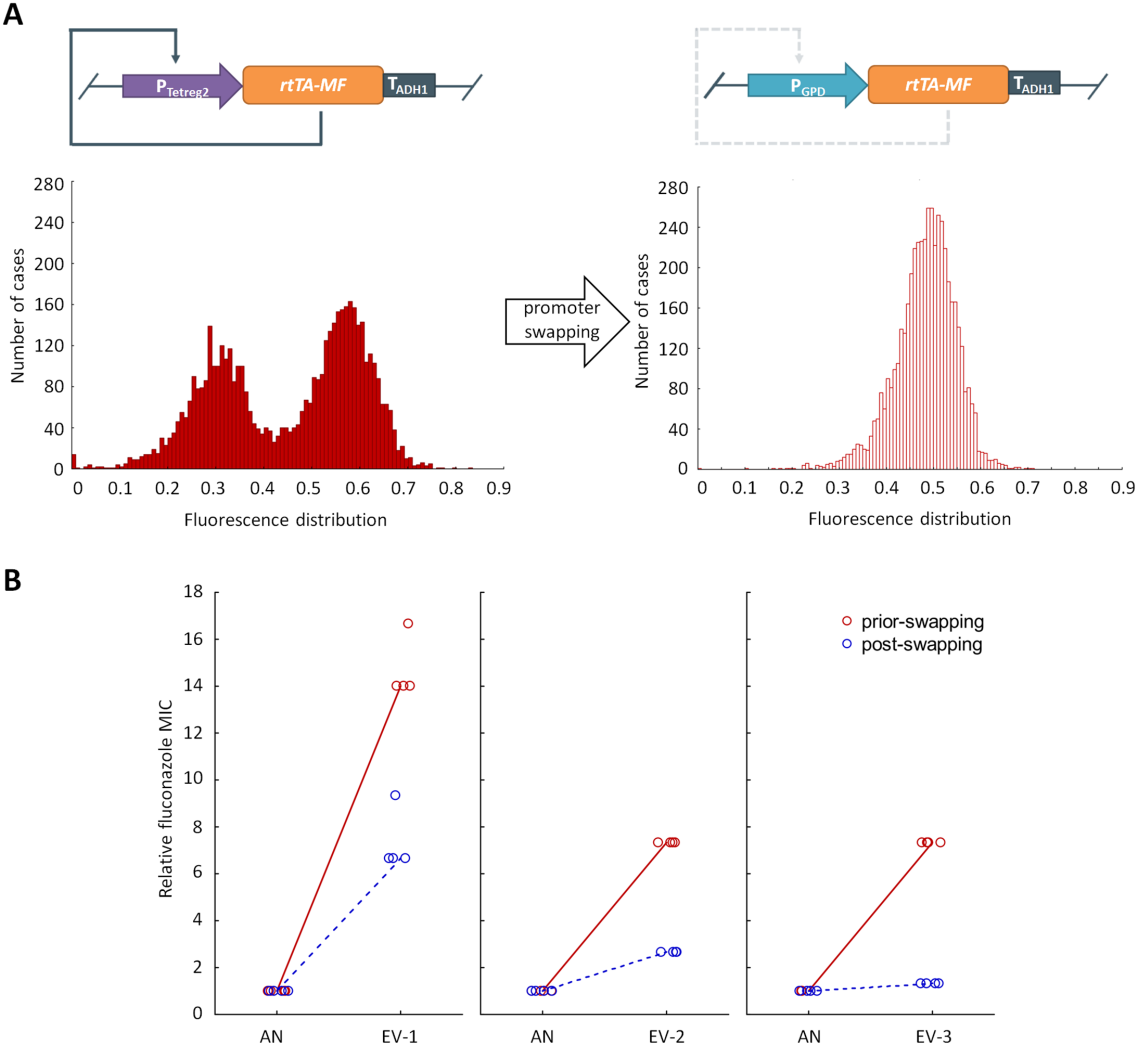
Outcome of the promoter-swap experiment. **(A) The promoter-swap experiment.** The promoter controlling the reverse-tetR trans-activator (*rtTA-MF*) gene in three selected evolved positive feedback (PF) strains was swapped for the corresponding promoter present in the ancestor no positive feedback (noPF) strain. As a result of eliminating the positive feedback loop (denoted as a dashed line), the PF evolved strains exhibited a C-terminally GFP-tagged Pdr5p protein (Pdr5p-GFP) fluorescence distribution reminiscent of the noPF strains. **(B) Evidence for synergism between adaptive mutations and phenotypic heterogeneity.** The figure shows the relative increase in the minimum inhibitory concentration (MIC) of three selected evolved PF strains (EV-1, EV-2 and EV-3), prior to (red full circle) and post (red empty circle) swapping. The median MIC values of the genotypes are normalized to that of the corresponding ancestor strains. After controlling for the level of MIC reduction in the ancestor strains, the promoter-swap procedure resulted in a 2.1–5.5-fold reduction in the resistance level of the evolved PF strains (Mann–Whitney *U* test, *p* < 0.05). Four independent cultures per strain were grown and measured for MIC (empty circles). The data underlying Fig 3 can be found in S1 Data.

Phenotypic heterogeneity may enlarge the phenotypic effects of mutations [32] and consequently increase the set of adaptive mutations that provide resistance above a critical fluconazole dosage. This prediction was first investigated by eliminating the positive regulatory feedback loop in three randomly selected, independently evolved strains from the final day of the experiments, all of which carried the PF genetic circuit (Fig 3A). Specifically, the promoter controlling the *rtTA-MF* gene was swapped for the corresponding promoter present in the ancestor noPF strain. The same procedure (promoter swap) was done with the corresponding ancestor PF strain. Elimination of the positive feedback loop left the mean *PDR5-GFP* expression unchanged but substantially reduced expression variability (i.e., the CV of expression, Fig 3A). As a result, fluconazole resistance level in the PF ancestor—estimated by minimum inhibitory concentrations—showed a relatively minor 2-fold reduction. By contrast, the same promoter-swap procedure in the evolved PF strains caused a 4.2–11-fold reduction in resistance level (Fig 3B). As the drop of resistance due to promoter swap was especially high in the evolved strains, we conclude that phenotypic heterogeneity has a large impact on the fitness effects of adaptive mutations that accumulated during the course of laboratory evolution.

**Fig 4:**
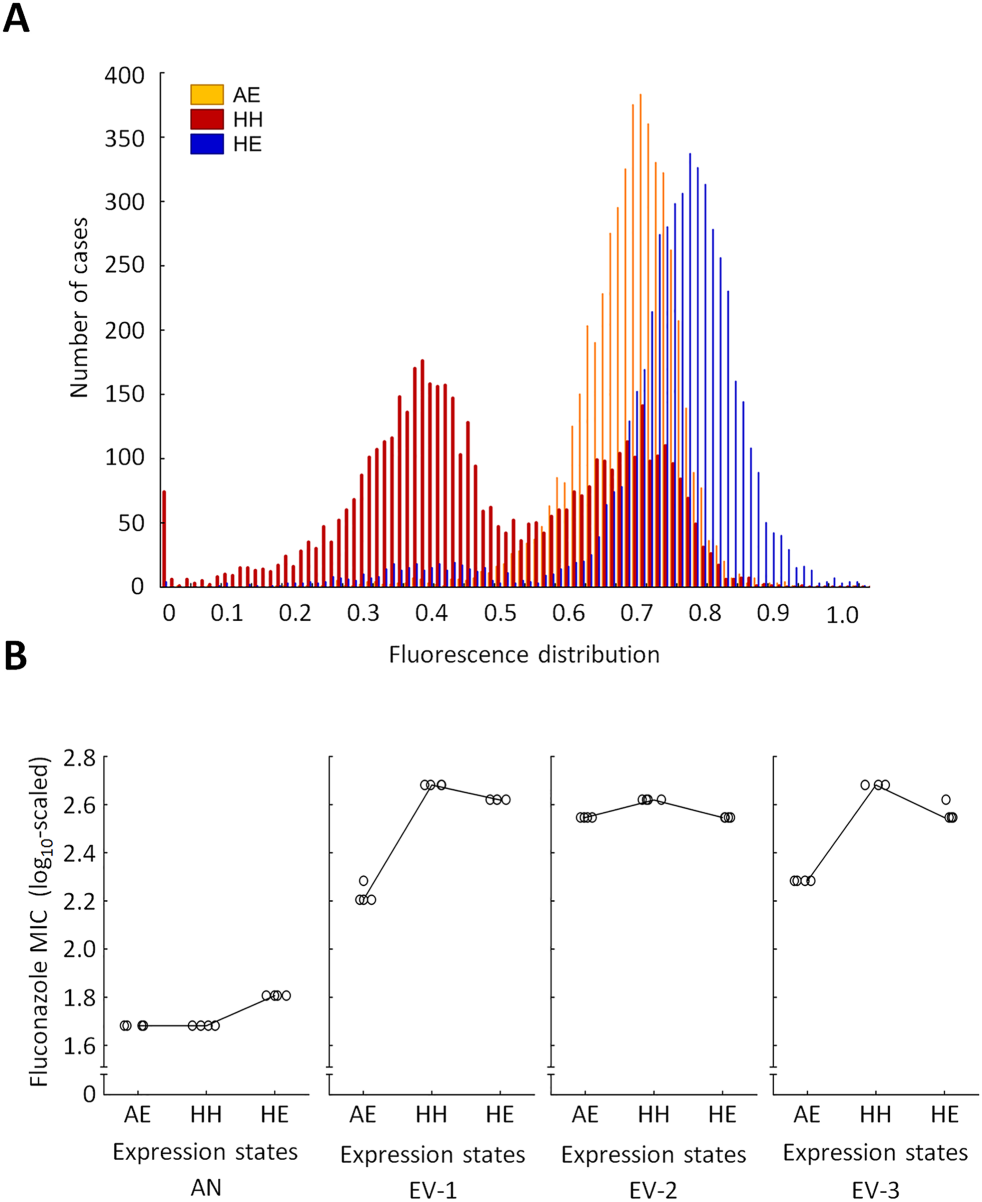
Phenotypic heterogeneity modulates the effect of mutations on resistance level. **(A) Distribution of C-terminally GFP-tagged Pdr5p protein (Pdr5p-GFP) fluorescence level at three different expression states by flow cytometry.** The histograms show the fluorescence distributions of Pdr5p-GFP, as follows: positive feedback (PF) with high phenotypic heterogeneity (HH), no positive feedback (noPF) with adjusted expression level (AE), and PF with high expression level (HE). The inducer levels were set to 0.2 μg/ml doxycycline (AE in noPF strain), 0.3 μg/ml doxycycline (HH in PF strain), and 3 μg/ml doxycycline (HE in PF strain), respectively. **(B) Comparison of HH, AE, and HE.** The figure shows the minimum inhibitory concentrations (MIC) of the ancestor (AN) and three evolved (EV) PF strains (EV-1, EV-2, and EV-3) in three different expression settings. The settings were HH, AE, and HE. In the ancestor, the AE and HH settings reached the same resistance level, as expected (Mann–Whitney *U* test, *p* = not significant), while the HE setting reached higher MIC level than HH (Mann–Whitney *U* test, *p* < 0.05). In the evolved strains (EV-1, EV-2, and EV-3), however, the MIC level of the HH setting was somewhat higher than that of AE (Mann–Whitney *U* test, *p* < 0.05) or HE (Mann–Whitney *U* test, *p* < 0.05). Four independent cultures per strain were grown and measured for MIC (empty circles). The data underlying Fig 4 can be found in S1 Data.

Next, we tested whether the advantage of phenotypic heterogeneity remains after controlling for differences in the initial fluconazole susceptibilities between PF and noPF. Careful adjustment of the inducer concentration (Fig 4A) in the noPF strain ensured that fluconazole resistance levels in the ancestor PF (high phenotypic heterogeneity [HH]) and noPF (adjusted expression level [AE]) strains are the same. (Fig 4B). In the evolved (EV) strains (EV-1, EV-2, and EV-3; Fig 4B), however, resistance level of the HH setting was somewhat higher than that of AE. This indicates that the advantage of high phenotypic heterogeneity remains when the PF and noPF have the same starting fluconazole susceptibilities. Finally, we note that high phenotypic heterogeneity is beneficial over low heterogeneity, high expression level (HE) setting in certain evolved strains (for details, see Fig 4B).

### Molecular mechanisms of resistance

**Fig 5:**
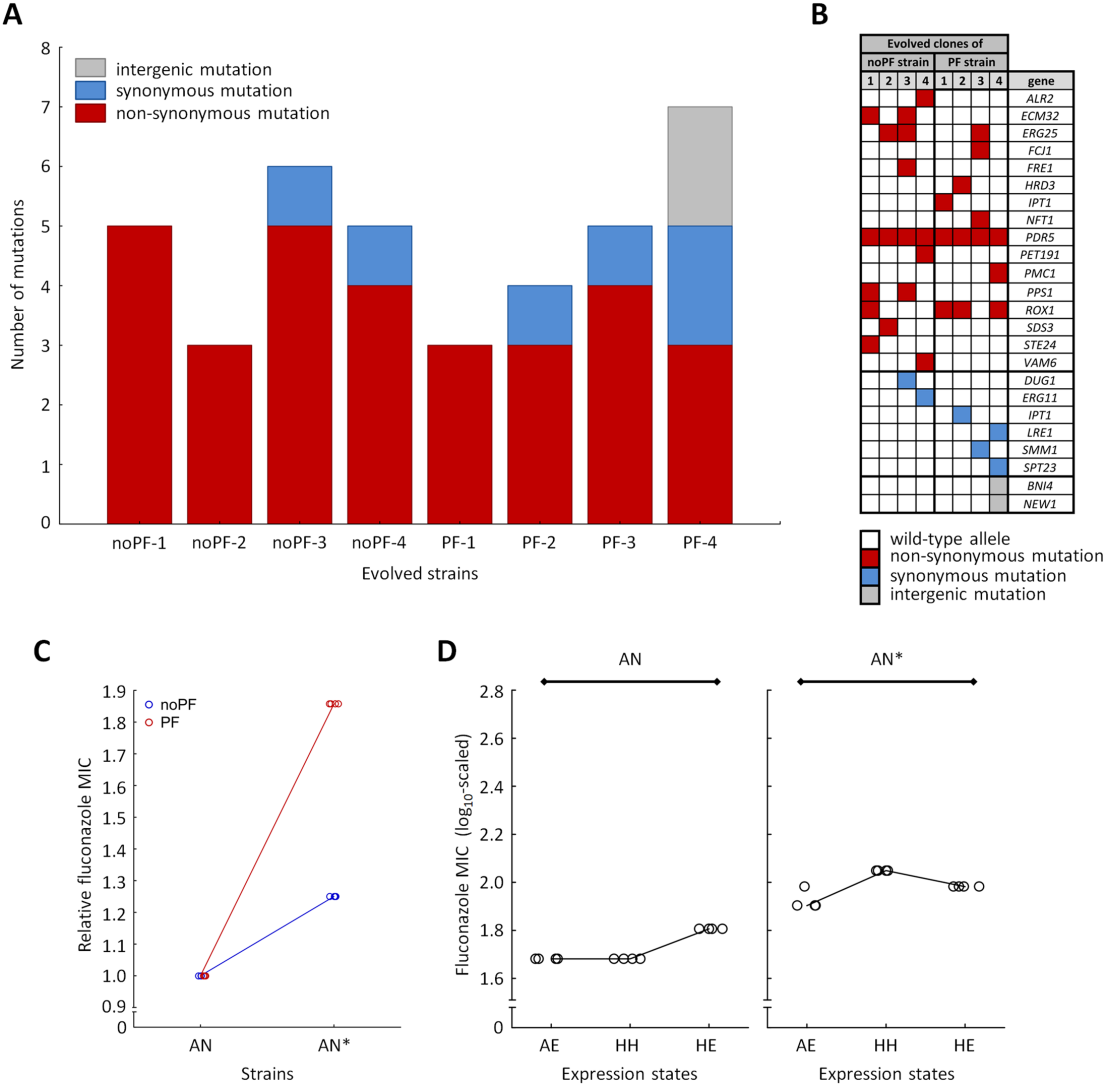
Genomic analysis. **(A) Distribution of mutational events across the evolved strains**. Four independently evolved positive feedback (PF) and four no positive feedback (noPF) strains were subjected to whole-genome sequencing, respectively. The figure shows the number and types of detected mutations per strain. There was no significant difference in the number of mutations between noPF and PF strains (Mann–Whitney *U* test, *p* = not significant). **(B) List of genes with nonsynonymous, synonymous, and intergenic mutations across the evolved strains**. As expected from the mode of action of fluconazole, many of the mutated genes are involved in ergosterol biosynthesis (*ERG25*) and regulation (*ROX1*) and membrane transport (*PDR5*, *NFT1*). **(C) Synergism between phenotypic heterogeneity and *PDR5* mutation**. The figure shows the relative fluconazole minimum inhibitory concentration (MIC) of the PF and noPF strains with a specific *PDR5* mutation (His595Asp) inserted. AN, ancestor; AN*, ancestor with a single *PDR5* mutation inserted. The median MIC values of the genotypes were normalized to that of the corresponding ancestor strains. Insertion of the mutation resulted in an 85% decline in fluconazole susceptibility when phenotypic heterogeneity was high, but its beneficial effect was reduced otherwise (Mann–Whitney *U* test, *p* < 0.05). Four independent cultures per strain were grown and measured for MIC (empty circles). **(D) Comparison of high phenotypic heterogeneity (HH), adjusted expression level (AE), and high expression level (HE) in a *PDR5* mutant strain.** The figure shows the MIC values of AN and AN* in three different expression settings. The expression settings were as follows: HH, AE, and HE. In the ancestor, the AE and HH settings reached the same resistance level, as expected (Mann–Whitney *U* test, *p* = not significant), while the HE setting reached higher MIC level than HH (Mann–Whitney *U* test, *p* < 0.05). In AN*, however, the MIC level of the HH setting was somewhat higher than that of AE (Mann–Whitney *U* test, *p* < 0.05) or HE (Mann–Whitney *U* test, *p* < 0.05). Four independent cultures per strain were grown and measured for MIC (empty circles). The data underlying Fig 5 can be found in S1 Data.

To gain insight into the molecular mechanisms of evolved resistance and its dependence on phenotypic heterogeneity, we randomly selected four independently evolved noPF and four independently evolved PF strains, respectively, from the final day of the Experiment A. Single clones of these strains were subjected to whole-genome sequencing using the Illumina Nextera XT protocol. As no large-scale genomic rearrangements were detected, we focused on analyzing point mutations only. In total, we observed 38 mutational events, 95% of which occurred in protein-coding regions (Fig 5A and S2 Table). Several observations indicate that the mutation accumulation in the protein-coding regions was largely driven by selection. More than eighty percent (83.3%) of these mutations were nonsynonymous, and many of them were found in genes with established links to fluconazole resistance (Fig 5B). Consistent with the hypothesis that fungicidal drugs (including azoles) induce oxidative damage [33], proteins with mitochondria- and respiration-related functions were frequently mutated. More generally, we found significant gene-level convergent evolution. As high as 22% of the mutated genes were shared by at least two strains, and some were shared extensively (Fig 5B). However, none of the mutations in different strains were identical at the nucleotide level, confirming that they accumulated independently of each other during the course of laboratory evolution. Repeatedly mutated genes include the natural multidrug transporter (*PDR5*, carried on the synthetic genetic circuit), sterol oxidase (*ERG25*), and a transcriptional regulator (*ROX1*) of ergosterol biosynthesis and respiration. The rarity of mutations in the molecular target of fluconazole (*ERG11*) under fluconazole stress has been observed previously [25] and may reflect high associated fitness cost of *ERG11* mutations or the efficiency of alternative resistance mechanisms.

To further investigate the impact of phenotypic heterogeneity on mutational effects, we focused on the multidrug transporter *PDR5*, not least because this gene was mutated in all of the sequenced strains. A randomly selected nonsynonymous mutation—observed in one of the evolved PF strains—was inserted individually into the ancestor strains (see Materials and methods) with PF and noPF genetic backgrounds, respectively (Fig 5C). The results confirmed the outcome of the promoter-swap experiments (Fig 3B). The mutation conferred a highly significant decline in fluconazole susceptibility when phenotypic heterogeneity was high, but its beneficial effect was substantially reduced otherwise (Fig 5C). Additional analyses confirmed that the effects of genomic mutations on resistance level were contingent on phenotypic heterogeneity (Figs 4B and 5D).

### Fitness costs of constitutively high gene expression

The above analysis leaves open the question of what the long-term advantage of phenotypic heterogeneity might be over mutations that simply provide a shift towards higher mean expression level. The key to this problem lies in the observation that high expression of a drug-resistance gene provides resistance, but it induces an especially high fitness cost in nonstressed conditions [28]. We hypothesized that the ultimate fate of elevated phenotypic heterogeneity should reflect a fundamental trade-off between the level of resistance and the fitness cost of resistance: compared to constitutively high expression level, phenotypic heterogeneity may dampen fitness costs when the level of fluconazole stress is relatively mild.

To investigate this issue experimentally, we focused on the ancestor and three randomly selected, independently evolved strains, all of which were carrying the PF genetic circuit (EV-1, EV-2 and EV-3). Pdr5p-GFP fluorescence distribution in all three strains remained unchanged during the course of laboratory evolution (S7 Fig). We investigated how modulation of *PDR5-GFP* mean expression level and simultaneous removal of gene expression stochasticity affect fitness under a wide range of fluconazole dosages. Careful adjustment of the inducer level allowed us to generate expression settings with low phenotypic heterogeneity but exceptionally high and low mean *PDR5-GFP* expression levels, respectively. The derived expression patterns were comparable to the low and high peaks of the PF strain, respectively (S8 Fig). We compared the fitness of each strain under low expression level (LE), high expression level (HE), and the original HH settings. This setup controls for the differences in the mutations that accumulated during the course of evolution and ensures direct comparison of the impact of *PDR5-GFP* expression patterns.

**Fig 6:**
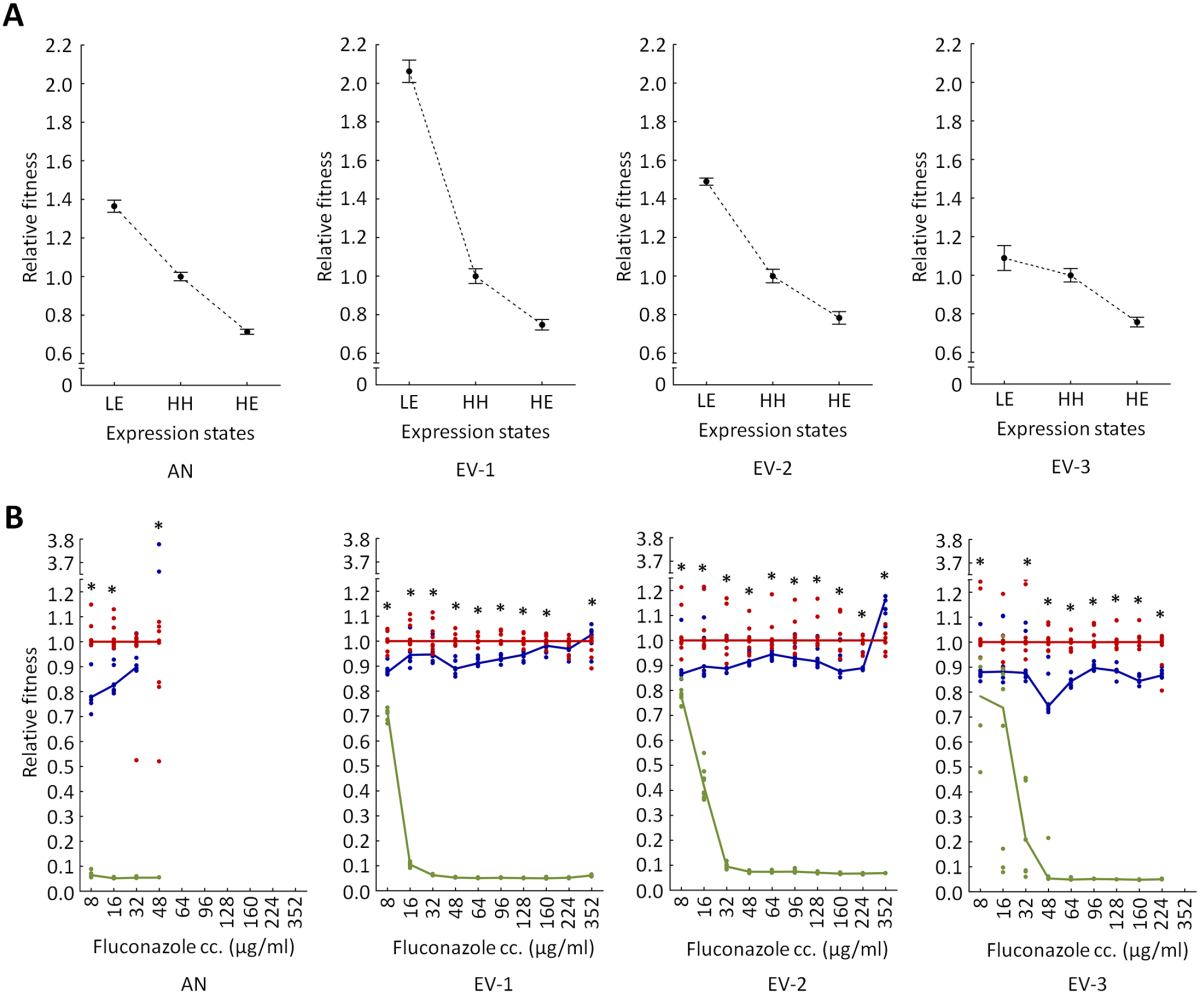
Comparison of phenotypic heterogeneity and constitutively high gene expression. **(A) High expression level (HE) of *PDR5* imposes a fitness cost.** The figure shows the mean relative fitness of four genotypes at different expression states in drug-free medium. The genotypes studied were the ancestor (AN) positive feedback (PF) and three evolved (EV) PF strains (EV-1, EV-2 and EV-3). The three expression states were as follows: low expression level (LE), HE, and the original high phenotypic heterogeneity (HH) setting (for further details see S8 Fig). Absolute fitness was estimated by the increment of the OD_600_ after 72 h of growth in drug-free medium. Relative fitness was calculated by normalizing to the absolute fitness of the corresponding strain in the HH expression state. Error bars indicate 95% confidence intervals, based on growth measurement of at least 60 independent cultures per expression state. (**B) Phenotypic heterogeneity provides fitness advantage under fluconazole stress**. The figure shows the relative fitness of four genotypes as a function of increasing fluconazole concentrations. The genotypes studied were the AN PF and three EV PF strains (EV-1, EV-2 and EV-3). Green line indicates LE, blue line indicates HE, while the red line indicates intermediate expression level with HH. Fitness was estimated by the OD_600_ values after 72 h of growth and was normalized to the fitness in the HH expression setting at each fluconazole dosage. The continuous lines connect the median relative fitnesses, based on growth measurement of eight independent cultures for each expression setting. * indicates significant fitness difference between HE and HH expression settings (Mann–Whitney *U* test, *p* < 0.05). In both panel A and B, the inducer levels were set to 0.015 μg/ml (LE), 0.3 μg/ml (HH), and 3 μg/ml (HE), respectively. For further details, see the main text. The data underlying Fig 6 can be found in S1 Data.

In the absence of fluconazole, the fitness in the HH setting was 28%–40% higher than the HE fitness, but 8%–52% lower than the LE fitness (Fig 6A). This indicates that selection ultimately favors low *PDR5-GFP* expression level (i.e., LE) in the absence of stress. The situation was more complex under fluconazole stress. Under a wide-range of fluconazole dosages, fitness in HH setting was higher than the corresponding HE and the LE fitnesses (Fig 6B). Remarkably, HH fitness was statistically higher or equal to the fitness in HE, even at the fluconazole dosage deployed during the final stage of laboratory evolution (224 μg/ml). As expected, fitness in LE was generally very low under most fluconazole dosages investigated (Fig 6B). Taken together, phenotypic heterogeneity appears to be favorable under a wide range of antifungal stress level compared to HE and LE settings. We suspect that this reflects an intricate balance between the level of resistance conferred and the fitness cost of resistance-bearing mutations. We will address this important issue in more detail in a future work.

## Discussion

Several studies indicate that phenotypic heterogeneity facilitates survival in dynamically changing environments, and promotes interactions and division of labor between individual cells [34]. It has also been suggested that stochastically generated phenotypes precede genetic changes and thereby facilitate rapid evolution of complex phenotypes [3,35]. This issue remained controversial due to the shortage of comprehensive tests from natural and experimental populations [35]. In this work, we combined synthetic biology, single-cell monitoring, and experimental evolution to explore the impact of phenotypic heterogeneity on evolvability. We developed two versions of an inducible synthetic gene circuit that generate varying degrees of expression stochasticity of an antifungal resistance gene.

The following main conclusions were reached. First, elevated phenotypic heterogeneity increased population survival under mild antifungal stress (Fig 1C), but, at the same time, it substantially reduced fitness in stress-free medium (Fig 1D). These results indicate a trade-off between gene expression costs and survival under stress conditions, which may shape phenotypic heterogeneity in nature. Second, phenotypic heterogeneity promoted adaptation towards increasing levels of antifungal stress. Populations with high phenotypic heterogeneity reached a higher level of resistance (Fig 2B) and were less likely to become extinct during the course of laboratory evolution (Fig 2A). Most importantly, we found no evidence that higher adaptation would reflect elevated local (S1 Table) or global (Fig 5A and S5 Fig) mutation rate associated with the PF genetic circuit. Third, the phenotypic effects of the mutations that accumulated during the course of laboratory evolution were contingent on phenotypic heterogeneity (Figs 3B and 5C). This result suggests that phenotypic heterogeneity may enlarge the set of adaptive mutations that provide resistance above a critical stress level. Finally, compared to constitutively high expression, phenotypic heterogeneity alleviated the fitness costs of target protein expression under a wide range of stress conditions. In sum, our study demonstrates that phenotypic heterogeneity promotes evolvability, partly by modulating the adaptive value of beneficial mutations. Several important predictions emerged from our study.

First, phenotypic heterogeneity should be especially favorable in deteriorating conditions. Indeed, the extent of phenotypic heterogeneity in wild-type yeast isolates is highest in stressful environments [36], indicating that heterogeneity facilitates adaptation to adverse conditions in the wild. Previous studies also indicate that genes are expressed when they are not needed for fitness [37]. Our work gives credit to the idea that many genes expressed in a certain fraction of the microbial populations are in a “standby mode”, and thereby help survival upon environmental change [38].

Second, our work focused on the evolutionary consequences of phenotypic heterogeneity and left open the issue of why phenotypic heterogeneity exists in nature. Phenotypic heterogeneity could generally be a by-product of the unavoidable imprecision of molecular processes. We note, however, that phenotypic heterogeneity can readily change in the laboratory (Fig 2C, see also [17]). Therefore, different forms of phenotypic heterogeneity in nature may evolve as a direct response to novel and extreme environmental challenges [39].

Our study also suggests that cell-to-cell phenotypic heterogeneity could initiate key steps of microbial drug resistance, for example by promoting fluctuations of protein concentrations in efflux pumps [40]. An important unresolved issue concerns the molecular mechanisms whereby gene expression noise shapes the beneficial effects of mutations in the protein (Fig 5C and 5D). This pattern may reflect a trade-off between protein stability and improved protein activity, as suggested previously [41]. Finally, future studies should reveal the extent by which phenotypic heterogeneity facilitates adaptive search for rare combinations of beneficial mutations, as seen in the case of key evolutionary innovations [10].

## Materials and methods

### Plasmid construction

To detect the level of Pdr5p (*YOR153W*, S000005679), the C-terminally *GFP*-tagged *PDR5* region (*PDR5-GFP*) was obtained from the available GFP collection [42] (Open BioSystem). The GFP-tagged Pdr5p remained fully functional. The *PDR5-GFP* was amplified by PCR using PDR5-BamHI-f and neGFP-XhoI-r primers. The resulting fragment was digested in two sequential reactions, as follows. First, it was divided into short upstream and long downstream fragments by using *Afl*II and *Xho*I restriction enzymes. In the second step, the short upstream fragment was also digested with *BamH*I enzyme. Afterwards, both the short upstream and the long downstream fragments were inserted into a *BamH*I-*Xho*I digested pDN-T2dGZmxh plasmid [22]. This resulted in the pDN-T2dPGxh reporter plasmid that contains the *PDR5-GFP* gene downstream of P_Tetreg2_, a modified version of P_CYC1_ containing two tetO2 sites. All cloning procedures were performed in *Escherichia coli* XL-10 Gold strain (Stratagene, La Jolla, CA), using ampicillin as selection marker (Sigma, St. Louis, MO). The inserted regions were verified by sequencing with double coverage. Sequences and description of the oligonucleotides used for strain construction can be found in S3 Table.

The regulatory plasmids (pDN-T2dMFot and pDN-GPMFot, for more details, see [22]) carry a modified version of the *rtTA-MF* that contains three minimal VP16 activator F domains. The expression of the *rtTA-MF* is either controlled by a synthetic tet-inducible tetreg promoter (P_Tetreg2_ in pDN-T2dMFot) or by a constitutive glyceraldehyde-3-phosphate dehydrogenase promoter (P_GPD_ in pDN-GPMFot). When the rtTAp is bound by an externally added inducer (doxycycline, Biochemica), it activates transcription by binding to the tetO2 sites in P_Tetreg2_.

### Yeast strain construction

All strains used in this study were derived from the YPH500 *S*. *cerevisiae* parental strain (α, *ura3-52*, *lys2-801*, *ade2-101*, *trp1Δ63*, *his3Δ200*, *leu2Δ1*) and were generated by yeast transformation using the standard lithium acetate procedure [43]. First, the pDN-T2dPGxh reporter plasmid (containing the *HIS3* selectable marker) was linearized with *Afe*I restriction enzyme and integrated into the *his3Δ200* locus of the YPH500 strain. As a result, an intermediate yeast strain, YDN-T2dPGxh was constructed. Transformants were selected on histidine drop-out synthetic medium (SC-His, 5 g/l ammonium sulfate, 1.7 g/l Yeast Nitrogen Base, supplemented by an amino acid mix without histidine). Second, the regulatory plasmids (pDN-T2dMFot and pDN-GPMFot, for more details, see [22]), containing the TRP1 selectable marker, were linearized with *Ahd*I restriction enzyme and integrated into the genome of the intermediate YDN-T2dPGxh yeast strain by using the *ampR* locus of the reporter construct for targeting. Transformants were selected on tryptophan drop-out synthetic medium (SC-Trp). As a result, YDN-T2dPGxh-T2dMFot (PF) and YDN-T2dPGxh-GPMFot (noPF) yeast strains were generated, respectively. Only strains with single genomic integrations of the constructs (verified by PCR) were used in this study.

In the PF strain, the *rtTA-MF* is expressed from the same P_Tetreg2_ as the *PDR5-GFP* gene. Therefore, the produced rtTAp induces the transcription of the *PDR5-GFP* and its own transcription, alike. This positive feedback loop generates high expression stochasticity of the *PDR5-GFP*. In the noPF strain, the same *rtTA-MF* is controlled by P_GPD_. Owing to absence of the positive feedback loop, the *PDR5-GFP* has low gene expression stochasticity.

Finally, the native *PDR5* gene was deleted from the genome of constructed PF and noPF strains as follows. The *PDR5*::*KanMX* deletion cassette was amplified with longer than 0.3 kb flanking region using the genomic DNA of the *Δpdr5* strain from the YKO Mat a collection [44] (Open Biosystem). This deletion cassette was transformed into the parental PF and noPF strains, and the correct transformants were selected on histidine and tryptophan drop-out synthetic medium (SC, 5 g/l ammonium sulfate, 1.7 g/l Yeast Nitrogen Base, supplemented by amino acid mix), containing G418 (Roche) selection drug at 200 mg/l final concentration.

Mutator strains were generated by inactivating a mismatch-repair gene (*MSH2*, S000005450) in PF and noPF strains, respectively. Briefly, the *MSH2*::*NatMX* deletion cassette was amplified using the genomic DNA of the *Δmsh2* strain from the SGA (Synthetic Genetic Array) query collection [45]. This deletion cassette was transformed into the PF and noPF strains, and the correct transformants were selected on SC–His/Trp containing nourseothricin (clonNAT, WERNER BioAgents) selection drug at 100 mg/l final concentration.

### Flow cytometry

An established flow cytometry protocol [22] was used to measure the distribution of steady-state gene expression level across cells from isogenic yeast cell populations. Briefly, single yeast colonies were picked from agar plates and incubated in synthetic drop-out medium supplemented with 2% glucose at 30°C. After reaching stationary phase, 1% of the population were serially transferred into fresh synthetic drop-out medium containing galactose as carbon source, supplemented with appropriate inducer concentrations: PF and noPF strains were induced by 0.3 μg/ml and 0.015 μg/ml doxycycline, respectively. After reaching stationary phase, 1% of the populations were serially transferred to fresh induction medium (synthetic drop-out medium with 2% galactose and inducer). The populations were grown until the stabilization of gene expression distributions (approximately 20 h). Cell suspensions were diluted to approximately 5 × 10^2^ cells/ml, and fluorescence intensity values (logarithmic scale) were estimated by a Guava flow cytometer. Gating was applied based on forward scatter data (logarithmic scale), to exclude extrinsic noise. During each run, a minimum of 5,000 events were recorded. The log_10_-scale fluorescence level of the cells was normalized to log_10_-scale forward scatter data (i.e., cell size). Phenotypic heterogeneity (CV) was computed for each population as the standard deviation normalized by the mean of fluorescence.

### Experimental evolution

Two complementary experiments were used to study the impact of phenotypic heterogeneity on adaptive evolution. Experiment A measured the extinction rate of the evolving populations as a function of gradually increasing fluconazole (Molekula) dosage, while experiment B aimed to maximize fluconazole resistance increment during a fixed time period.

Experiment A was conducted in 96-well plates, using 42 independent populations of noPF and 42 independent populations of PF strains, respectively (S2A Fig). The populations were subjected to parallel laboratory evolution in histidine and tryptophan drop-out synthetic medium (SC-Trp/His) containing doxycycline to induce the synthetic genetic circuits in 96-well deep-well plates (0.5 ml, polypropylene, V-bottom). Plates were covered with sandwich covers (Enzyscreeen BV) to ensure an optimal oxygen exchange rate and limit evaporation, shaken at 280 rpm, and incubated at 30°C. Ten percent of the populations were serially transferred into 350 μl of fresh medium every 72 h. The relatively long time period between transfers and the increased medium volume (i.e., increased population size) was necessary, as growth rates of the evolving populations were low at high fluconazole dosages. Starting at a subinhibitory fluconazole concentration (8 μg/ml), fluconazole dosage was increased gradually at every second transfer. The applied dosages were as follows: 0, 8, 16, 24, 32, 64, 96, 128, 160, 192, and finally 224 μg/ml. Samples from each time interval, including time-zero, were frozen in 15% glycerol and kept at −80°C until fitness measurement or further analysis. Cross-contamination events were regularly checked by visual inspection of blank wells containing only medium. Doxycycline and fluconazole stock solutions were made fresh before the transfer by dissolving powder stocks in specified solvents by the manufacturer’s instructions. Population extinction was defined by a cutoff OD_600_ = 0.15 after 72 h of cultivation. Evolved strains from the final day of experiment A were used for further genomic and functional analyses.

Experiment B followed the protocol of an established automated evolution experiment [46]. Ten independent populations each of both noPF and PF strains were propagated in parallel, in the same medium and culturing conditions as in Experiment A. A checker board layout was used on the plate to monitor cross-contamination events (S2B Fig). Starting with the subinhibitory drug concentration, each culture was allowed to grow for 72 h. Twenty μl of culture was transferred to four independent wells containing fresh medium and increasing dosages of fluconazole (0.5x, 1x, 1.5x, and 2.5x the concentration of the previous step). At each transfer, cell growth was monitored by measuring the optical density at 600 nm (OD_600_ value, Biotek Synergy 2 microplate reader was used for this purpose). Only populations with (a) vigorous growth (i.e., OD_600_ > 0.2) and (b) the highest drug concentration were selected for further evolution. Accordingly, only one of the four populations was retained for each independently evolving strain. This protocol was designed to avoid population extinction and to ensure that populations with the highest level of resistance were propagated further during evolution.

### Evaluation of population size

To estimate population size from raw OD_600_ values, an established protocol [47] was used to correct for the nonlinearity of OD measurements in high-density cultures. Corrected OD values were then converted to cell number according to established yeast protocols (i.e., OD_600_ = 1 is equal to 3 × 10^7^ cells/ml, [48]).

### Evaluation of mutation rate

Can^R^ (canavanine resistance) spontaneous mutation rate was estimated by performing a standard fluctuation assay, as previously described [49]. Briefly, a stationary culture inoculated from a single colony was diluted to an approximately 10^2^-cells/ml density and separated into six independent cultures. The independent cultures were incubated until early stationary phase then appropriate dilutions were spread onto nonselective YPD solid medium as well as SC arginine-dropout solid medium containing 60 mg/liter canavanine. After 3 days of incubation at 30°C, colonies were counted. The mutation rate was calculated using the Lea-Coulson Method of the Median [50]. The calculations were performed using the FALCOR web tool [51].

### MIC determination

MIC values were determined using a standard linear broth dilution technique [52] in 96‐well microtiter plates (Sarstedt). Approximately 10^4^ to 10^5^ yeast cells were inoculated into each well of the 96-well microtiter plates (containing varying concentrations of fluconazole) with a 96‐pin replicator (VP407, V&P Scientific) and were propagated at 30°C shaken at 280 rpm. After 72 h of incubation, raw OD_600_ values were measured in a Biotek Synergy 2 microplate reader (BioTek Instruments). MIC was defined by a cutoff OD_600_ value (0.15).

### High-throughput fitness measurements

Established protocols [53] were used to measure the fitness of the yeast populations without fluconazole stress. Growth was assayed by monitoring the OD_600_ value of liquid cultures of each strain using 384-well density microtiter plates.

The prestarter cultures were inoculated from frozen samples into a medium containing 2% glucose using a VP407 replicator (V&P Scientific). The prestarter plates were incubated at 30°C in a rotary shaker. After reaching stationary phase, the prestarter plates were replicated into a medium containing 2% galactose and appropriate concentrations of doxycycline (Biochemica). After reaching the stationary phase, 384-well density plates—filled with 60 μl inducing SC–His/Trp medium per well—were inoculated for growth curve recording using a pin tool with 1.58 mm floating pins. The pin tool was moved by a Microlab Starlet liquid handling workstation (Hamilton Bonaduz AG) to provide uniform inocula across all samples. Cultures were incubated at 30°C in an STX44 19 (LiCONiC AG) automated incubator with shaking speed alternating every minute between 1,000 rpm and 1,200 rpm. Plates were transferred by a Microlab Swap 420 robotic arm (Hamilton Bonaduz AG) to Powerwave XS2 plate readers (BioTek Instruments) every 20 min, and cell growth was followed by recording the OD_600_ value. To estimate fitness, the increment of the OD_600_ was calculated from the obtained growth curves, following established procedures [54,55]: the average of the initial five OD_600_ values was subtracted from the last OD_600_ value of the corresponding curve. All values were blank corrected, OD calibrated, and smoothed by averaging and by removing negative slopes.

### Promoter-swap

To determine the impact of gene expression stochasticity on the acquired resistance of the evolved PF strains, the promoter that controls the expression of *PDR5*-*GFP* was cross-swapped with the promoter of the noPF strain. A double-joint PCR method [56] was applied for this purpose. First, a selectable auxotrophic marker (*CaUra3*, component M) was amplified by PCR with chimeric primers that contain complementary overhangs to the flanking regions of the promoter. The 5′ and 3′ flanking regions of the *rtTA-MF* transactivator gene promoters (component 5′ flanking and component 3′ flanking, respectively) were amplified and joined to the selectable marker by nested PCR. The joint cassettes were transformed into the evolved strains, and transformants were selected on uracil drop-out synthetic medium. The correct transformants were confirmed by PCR using primers designed to the flanking regions. The consequent change in gene expression stochasticity was confirmed by flow cytometry, as described above. Sequences and descriptions of the oligonucleotides used for promoter-swap can be found in S4 Table.

### Whole-genome sequencing

To reveal the underlying molecular mechanisms of acquired resistance, four independently evolved PF and four independently evolved noPF strains were subjected to whole-genome sequencing, respectively. Single clones were picked and isolated by streaking the evolved populations onto solid medium. Fitness increase and Pdr5p-GFP fluorescence distribution of the clones were retested after isolation. Only confirmed representatives of the corresponding evolved populations were further investigated.

Genomic DNA was prepared using a glass bead lysis protocol: clones were inoculated into 2 ml SC–His/Trp and grown to saturation at 30°C. Cells were pelleted and resuspended in 500 μl of lysis buffer (1% SDS, 50 mM EDTA, 100 mM Tris pH 8). Cells were mechanically disrupted by vortexing for 3 min at high speed with 500 μl glass bead (500 μm, acid washed). The proteins and other contaminants are precipitated from the crude cell lysate using high concentrations of ammonium acetate (275 μl 7 M ammonium acetate, final concentration: 2.5 M). This salting-out step was performed at 65°C for 5 min. To separate the nucleic acids from the contaminants, an organic extraction method was used. After cooling the samples for 5 min, an equivalent volume of chloroform:isoamyl-alcohol (24:1) was added. After centrifugation for 10 min, the nucleic acid containing aqueous layer was transferred into a new tube and precipitated with an equivalent volume of 1 ml isopropanol. The nucleic acid was pelleted and washed with 70% ethanol and resuspended in 500 μl RNaseA solution (50 ng/ml). After 30 min RNaseA treatment at room temperature, samples were chloroform:isoamyl-alcohol (24:1) extracted, precipitated with 50 μl sodium acetate (3 M pH 5.2) and 1,250 μl ethanol, pelleted, and washed with 70% ethanol. Finally, the genomic DNA (gDNA) was dissolved in 10 mM Tris-HCl (pH 8–8.5).

Genomic DNA was quantified using a Qubit (Invitrogen) 2.0 fluorometer with a Qubit dsDNA HS Assay. Starting from the recommended 1 ng of gDNA, the samples were processed according to Illumina Nextera XT protocol, with the exception that the bead-normalization step was replaced by library quantification using a qPCR assay KAPA Library Quantification kit. Library fragment distribution was analyzed by Bioanalyzer (Agilent) using a High Sensitivity DNA chip. All libraries were normalized to 4 nM and pooled. The resulting final library was denatured and diluted before loading on a MiSeq cartridge (Illumina MiSeq Reagent Kit v3) for a paired-end 2 x 300 bp run (90x coverage). Raw sequencing data can be obtained from the European Nucleotide Archive (ENA) with the following accession number: PRJEB9425 (URL: http://www.ebi.ac.uk/ena/data/view/PRJEB9425).

### Bioinformatic analysis

Raw sequence data of paired end reads were filtered according to length and read quality. The reads were trimmed by removing consecutive bases on both 5′ and 3′ flanks with base quality less than 20. Thereafter, reads were filtered for length (minimum of 50 bp), ambiguity (N bases content <5%), and Average Quality Filter (minimum of 20). All reads not complying with these criteria were discarded.

Remaining paired reads were then mapped to the reference *S*. *cerevisiae* genome obtained from the Saccharomyces Genome Database, using the BWA Read Mapper application [57]. All mapping options of BWA were set using default values. The GATK pipeline [58], with default parameters, was used for indel calling and to realign reads around indels.

After mapping, another stage of reads filtering was performed to remove reads that interfered with the accuracy of the final results. Thus, unmapped, improperly aligned, and duplicated reads were removed using SAMtools [59]. In addition, reads with low mapping quality (less than 20) and reads with less than 95% of sequence identity to the reference were also discarded. Finally, reads with low or high insert size were filtered as well. To define both the lower and higher thresholds for which the value of an insert size should stand, the mean and standard deviation of that value for all the remaining read pairs was calculated, and reads with lower or higher insert size than the mean minus/plus the standard deviation were discarded, respectively. This process was repeated for each sample.

After the reads mapping and filtering process, variant calling was performed to identify single-nucleotide polymorphisms (SNPs) and copy-number variations (CNVs). Identification of potential CNVs was detected using the CNV-seq application and default parameters [60]: no large-scale CNV was observed in the evolved strains relative to their corresponding ancestors.

For the identification of SNPs, the SAMtools Variant Calling application was used [59]. All the Genotype Likelihood options and filter options provided by the application were set using the default values. SAMtools computes the likelihood of having a SNP in each genomic position. After SNP calling, genomic regions not well covered by the reference assembly were identified, and SNPs detected in those regions were discarded (e.g., fewer than ten reads). Additionally, SNPs positioned in repeated genomic regions, as determined by RepeatMasker [61] using default parameters and the reference genome, were also discarded from the final dataset. Up to this point, the SNP calling process was performed in order to find variations regarding the genome reference used in the mapping process. Last, the SNP list of the evolved and ancestor strains were compared to identify mutations that accumulated during the course of laboratory evolution. Genomic single-nucleotide polymorphisms with less than 100 variant quality score (Phred-scaled) or lower than 0.9 mutant/reference ratio were ignored.

### CRISPR-Cas9–mediated allele replacement

A mutation in *PDR5* conferring His595Asp amino acid change was generated by CRISPR-Cas9–mediated genome engineering, using an established workflow [62]. The targeting CRISPR plasmid was constructed by replacing the *CAN1* targeting gRNA construct on p426-SNR52p-gRNA.CAN1.Y-SUP4t with the corresponding *PDR5*-specific gRNA sequence by whole-plasmid amplification and subsequent ligation. Briefly, primers carrying the target region of His595 of the *PDR5* gene were used to PCR amplify the CRISPR plasmid. Subsequent recircularization of the PCR product produced the desired *PDR5*-targeting construct, containing a yeast selectable marker (*URA3*). Correct clones were verified by colony-PCR and sequencing with pYEST_frw and pYEST_rev primers. dsDNA donor cassette, carrying the desired SNV (single nucleotide variation), was generated by annealing equimolar amounts of single-stranded oligonucleotides (H595DF and H595DR, all purchased from Integrated DNA Technologies) by first denaturing the mixture of the two complementary strands in 50 mM NaCl at 95°C for 5 min and then allowing the samples to cool down to room temperature for 2 h.

Introduction of the desired SNP was carried out in two steps. First, a Cas9 expression plasmid (p415-GalL-Cas9-CYC1t) was transformed into the corresponding ancestral strains using a standard transformation method [43]. Transformants were selected on leucine drop-out synthetic complete medium. To induce Cas9 expression, cells carrying p415-GalL-Cas9-CYC1t were grown until saturation in 10 ml selection medium, containing 1% raffinose as carbon source and 2% galactose as inducer, then diluted to OD_600_ = 0.3 in the same medium. Cells from the exponential phase were used for electrocompetent cell preparation [63] for the second step. The CRISPR-Cas9–stimulated homologous recombination was carried out by the coelectroporation of the corresponding p426-SNR52p-gRNA-SUP4t plasmid (300 ng) and SNV-incorporating donor dsDNA (50 μg) into electrocompetent cells expressing Cas9. Electroporated cells were then transferred from each cuvette into 8 ml of a 1:1 mix of 1M sorbitol/YPD medium and allowed to recover at 30°C for 1 h. The cells were then diluted 100-fold and inoculated into selection medium (leucine and uracil drop-out SC medium) containing 1% raffinose and 2% galactose and incubated at 30°C on a rotary shaker (280 rpm) for 48 h. Upon reaching stationary phase, cells were subcultured twice in the same medium as before. Cells were then diluted and plated onto solid selection plates and allowed to grow at 30°C until colonies appeared. Correct clones were verified by yeast allele-specific colony-PCR. PCR products were assayed by agarose gel electrophoresis. Mutations were also confirmed by capillary sequencing of the corresponding *PDR5* genomic region. Sequences and descriptions of the oligonucleotides used in in CRISPR-Cas9–mediated allele replacement can be found in S5 Table.

## Supporting information

S1 Fig Distribution of PDR5-GFP fluorescence level across different levels of inducer concentration. The histograms show the fluorescence distributions of PDR5-GFP for PF ancestor as a function of increasing doxycyclin concentrations. Unimodal distribution was present until 0.075 μg/ml doxycycline and from 3 μg/ml doxycycline. Bimodal expression emerged at 0.15 μg/ml doxycycline and lasts till 1.2 μg/ml doxycycline. The data underlying S1 Fig can be found in S1 Data.
(TIF)

S2 Fig Concept figure of the laboratory evolution protocols. Concept figure of the laboratory evolution protocols. (A) Experiment A measured the extinction rate of the evolving populations as a function of gradually increasing fluconazole dosage. The concept figure shows the basic steps of Experiment A. At the first step, 42–42 independent populations of noPF and PF strains were set up (color-coded as green wells), respectively. The noPF and PF populations were evolved parallel on different 96 well plates. Checker board layout was used on the plate to monitor cross-contamination events. Blank wells are color-coded as black wells. 10% of the populations were serially transferred into fresh medium every 72 hours. Starting at a sub-inhibitory fluconazole concentration (step 2., 8 μg/ml), fluconazole dosage was increased gradually at every second transfer (e.g. at step 4., after applying the same concentration as at step 3.). The applied dosages were as follows: 0, 8, 16, 24, 32, 64, 96, 128, 160, 192 and finally 224 μg/ml. Population extinction (color-coded as white wells) was defined by a cut-off OD600 = 0.15 after 72 hours of cultivation. Evolved strains from the final day of Experiment A (step n.; after reaching resistance towards 224 μg/ml fluconazole) were used for further genomic and functional analyses. (B) Experiment B aimed to maximize fluconazole resistance increment during a fixed time period. The concept figure shows the basic steps of Experiment B. The same medium and culturing conditions were used as in Experiment A, using the same checker board layout (blank wells are color-coded as black). For simplified representation purposes, only the A2, A4, A6 and A8 wells of the microtiter plate are considered here as evolving populations. Growing populations are color-coded as green wells, whereas populations before growth or populations that did not grow are color-coded as faint green. Starting with sub-inhibitory drug concentration (step 1.), each population was allowed to grow for 72 hours. Populations were transferred (step 2.) to four independent wells (i.e. into the same position on four independent microtiter plates) containing fresh medium and increasing dosages of fluconazole (0.5x, 1x, 1.5x and 2.5x the concentration of the previous step). After 72 hours of incubation, cell growth was monitored (step 3.) as previously. Only populations with a) vigorous growth (i.e. OD600 > 0.2) and b) the highest drug concentration were selected for further evolution. Accordingly, only one of the four populations was retained for each independently evolving strain to make a transfer plate (step 4.). The transfer plate was used to inoculate four new microtiter plates (step 5.), each containing different fluconazole concentration (0.5x, 1x, 1.5x and 2.5x the concentration of the previous step) for each independently evolving populations, respectively. Steps 2–4. were repeated multiple times, until evolving population reached clinically relevant fluconazole resistance. The adaptation rate towards fluconazole was compared between the noPF and PF strains.
(TIF)

S3 Fig Outcome of Experiment B. The figure shows the resistance level of ten independent populations of noPF (blue circles) and ten independent populations of PF (red circles) strains, as a function of time (transferring steps). The data underlying S3 Fig can be found in S1 Data.
(TIF)

S4 Fig Comparison of noPF and PF population size throughout Experiment B. The figure shows the population size (cells/ml) of noPF and PF strains (after 72 hours of growth) as a function of transferring steps. We found no significant difference in the population size between evolving PF and noPF populations (paired t-test, P = N.S.). See Materials and methods for evaluation of population size. The data underlying S4 Fig can be found in S1 Data.
(TIF)

S5 Fig Comparison of noPF and PF mutation rate. The boxplot shows the mutation rates of non-mutator and mutator noPF (red) and PF strains (blue), respectively. The box shows the upper and lower quartiles, the black line the median (based on six replicates), and the whiskers the non-outlier range. There was no significant difference in the mutation rate between non-mutator PF and non-mutator noPF strains (t-test, P = not significant), and between mutator PF and mutator noPF strains (t-test, P = not significant). This result is consistent with the results of whole genome sequence analysis of the evolved strains: There was no significant difference in the number of mutations between noPF and PF strains (Fig 5A, main text). The mutator strains show an average 10-fold increase in mutation rate relative to their corresponding non-mutator strains. See Materials and methods for evaluation of population size. The data underlying S5 Fig can be found in S1 Data.
(TIF)

S6 Fig Distribution of PDR5-GFP fluorescence level in the evolved noPF strains. (A) The histograms show the fluorescence distributions of PDR5-GFP in noPF evolved strains (blue, noPF1-26), noPF ancestor (blue, noPF AN) and PF ancestor (red, PF AN). (B) Deviation of the fluorescence distributions from the normal distribution. The barplot shows the deviation (D value) of the fluorescent distributions in PF ancestor (PF AN, red), noPF ancestor (noPF AN, blue) and noPF evolved strains (noPF EV1-26, blue). The larger the D value is, the larger the deviation from the expected normal distribution. To calculate D value, a standard Kolmogorov-Smirnov normality test was performed. The data underlying S6 Fig can be found in S1 Data.
(TIF)

S7 Fig Distribution of PDR5-GFP fluorescence level in evolved PF strains. The histograms show the fluorescence distributions of PDR5-GFP in three representative evolved PF strains (red, PF EV-1-3) and PF ancestor (black, PF AN). The data underlying S7 Fig can be found in S1 Data.
(TIF)

S8 Fig Distribution of PDR5-GFP fluorescence level at three different expression states by flow cytometry. The histograms show the fluorescence distributions of PDR5-GFP for the ancestor PF strain at three different expression states: low expression level (LE, induced by 0.015 μg/ml doxycyclin), high heterogeneity (HH, induced by 0.3 μg/ml doxycyclin) and high expression level (HE, induced by 3 μg/ml doxycyclin). Both the mean of expression and coefficient of variation differed in HE and LE state, compared to the HH state: the mean was higher by 54% for HE (Mann Whitney U-test, P < 0.001) and lower by 20% for LE (Mann Whitney U-test, P < 0.001), while the coefficient of variation was lower by 52% and 57%, respectively (Mann Whitney U-test, P < 0.001). The data underlying S8 Fig can be found in S1 Data.
(TIF)

S1 Table Mutations observed in the genetic circuit. We isolated 28–28 independently evolved noPF and PF strains, respectively. The noPF strains displayed substantial increase in the coefficient of variation (CV), see Fig 2C in the main text. We hypothesized that mutations accumulated during the course of laboratory evolution in the reverse tetR coding sequence (rtTA-MF) of the genetic circuit, as this region has a key role in modulating PDR5 expression stochasticity. To decipher the molecular underpinnings of the changes in expression distribution, we sequenced the reverse tetR coding sequence (rtTA-MF) in the evolved strains, using Sanger sequencing. In agreement with expectation, 25% of the evolved noPF strains displayed a mutation in this region, while the reverse tetR mutant counts were as low as 3.3% in the PF strains (chi-squared test P < 0.05).
(XLSX)

S2 Table Results of the whole genome sequencing. (XLSX)

S3 Table Oligonucleotides used for cloning, sequencing and yeast strain validation. (XLSX)

S4 Table Oligonucleotides used in promoter-swap experiment. (XLSX)

S5 Table Oligonucleotides used in CRISPR-Cas9 mediated allele replacement. (XLSX)

S1 Data All data used to create figures in the manuscript. The file contains the datasets used to create the main and supplementary figures.
(XLSX)

## Abbreviations

AE: adjusted expression level
AN*: ancestor with a single *PDR5* mutation inserted
AN: ancestor
CV: coefficient of variation
dox: doxycycline
EV: evolved
HE: high expression level
HH: high phenotypic heterogeneity
LE: low expression level
MIC: minimum inhibitory concentration
noPF: no positive feedback
OD_600_: optical density at 600 nm
PDR5-GFP: C-terminally *GFP*-tagged *PDR5* gene
PDR5p-GFP: C-terminally GFP-tagged Pdr5p protein
PF: positive feedback
P_GPD_: glyceraldehyde-3-phosphate dehydrogenase promoter
P_Tetreg2_: synthetic tet-inducible tetreg promoter
rtTA-MF: reverse-tetR trans-activator gene
rtTAp: reverse-tetR trans-activator protein
t_*ADH1*_: terminator sequence of alcohol dehydrogenase 1
t_*CYC1*_: terminator sequence of cytochrome c
TDH3: glyceraldehyde-3-phosphate dehydrogenase
