# Correction: Phenotypic heterogeneity promotes adaptive evolution

**DOI:** 10.1371/journal.pbio.1002607

**Published:** 2017-06-20

**Authors:** Zoltán Bódi, Zoltán Farkas, Dmitry Nevozhay, Dorottya Kalapis, Viktória Lázár, Bálint Csörgő, Ákos Nyerges, Béla Szamecz, Gergely Fekete, Balázs Papp, Hugo Araújo, José L. Oliveira, Gabriela Moura, Manuel A. S. Santos, Tamás Székely, Gábor Balázsi, Csaba Pál

The following information is missing from the Funding section:

Dr. Rollin D. Hotchkiss Foundation. Received by ZB. NIH MIRA https://grants.nih.gov/grants/guide/rfa-files/RFA-GM-17-002.html (grant number R35GM122561). Received by GB. Aveiro Institute of Biomedicine—iBiMED www.ua.pt/ibimed (Research Unit N° UID/BIM/04501/2013, Project Reference: PTDC/BEX-BCM/2121/2014). Received by MASS. Portuguese Foundation for Science and Technology (FCT) www.fct.pt and the European Structural funds (FEDER). Received by iBiMED and MASS. The funders had no role in study design, data collection and analysis, decision to publish, or preparation of the manuscript.

## References

[1] Bódi Z, Farkas Z, Nevozhay D, Kalapis D, Lázár V, Csörgő B, et al (2017) Phenotypic heterogeneity promotes adaptive evolution. PLoS Biol 15(5): e2000644 https://doi.org/10.1371/journal.pbio.2000644 28486496 10.1371/journal.pbio.2000644 PMC5423553

