# Supplementary figures and images for: Phenotypic heterogeneity promotes adaptive evolution
Source: PLoS Biol. 2017 May 9;15(5):e2000644. doi: 10.1371/journal.pbio.2000644 (PMC5423553; doi:10.1371/journal.pbio.2000644)

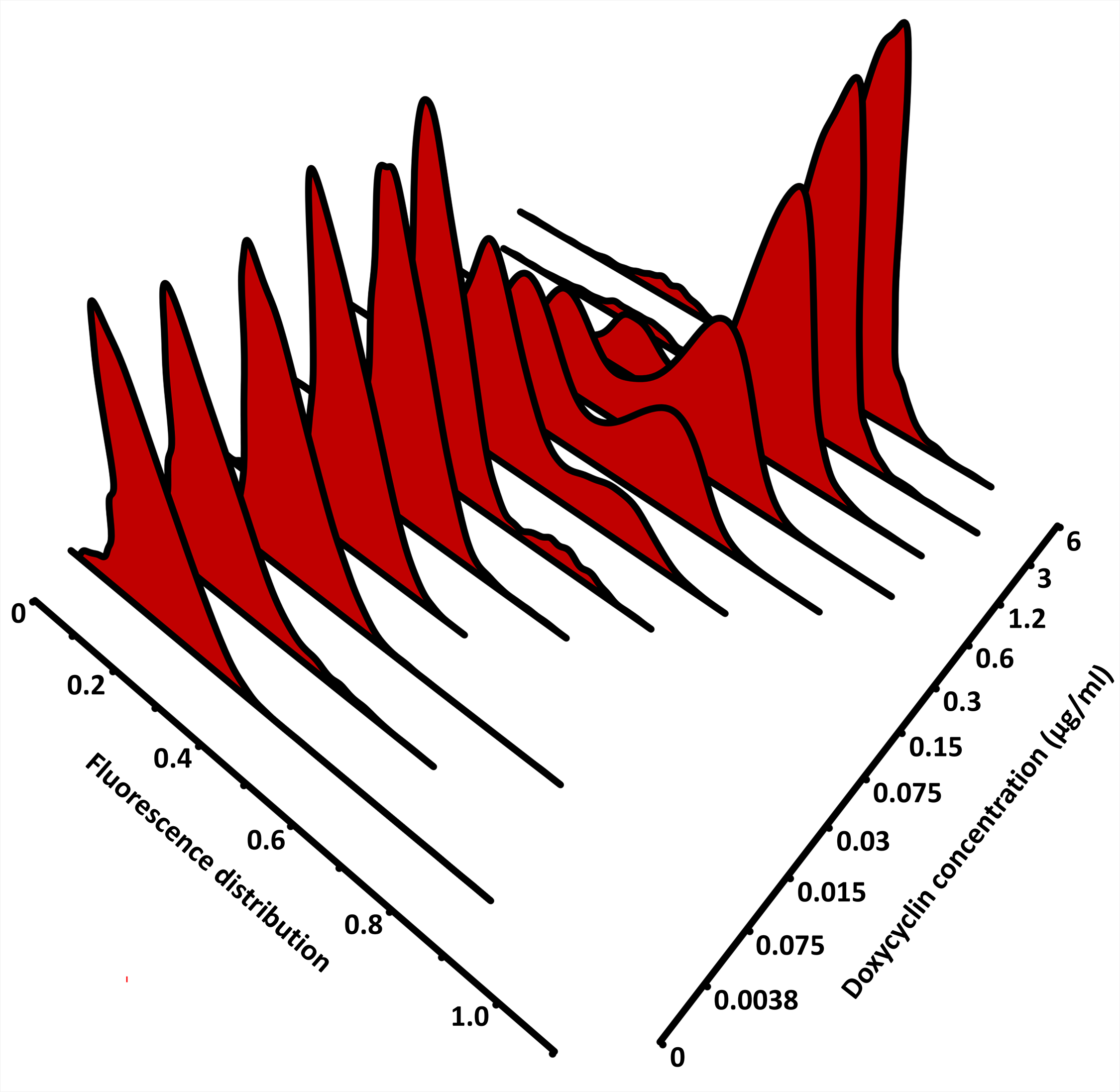

Supplement: S1 Fig — The histograms show the fluorescence distributions of PDR5-GFP for PF ancestor as a function of increasing doxycyclin concentrations. Unimodal distribution was present until 0.075 μg/ml doxycycline and from 3 μg/ml doxycycline. Bimodal expression emerged at 0.15 μg/ml doxycycline and lasts till 1.2 μg/ml doxycycline. The data underlying S1 Fig can be found in S1 Data. (TIF) [file pbio.2000644.s001.tif]

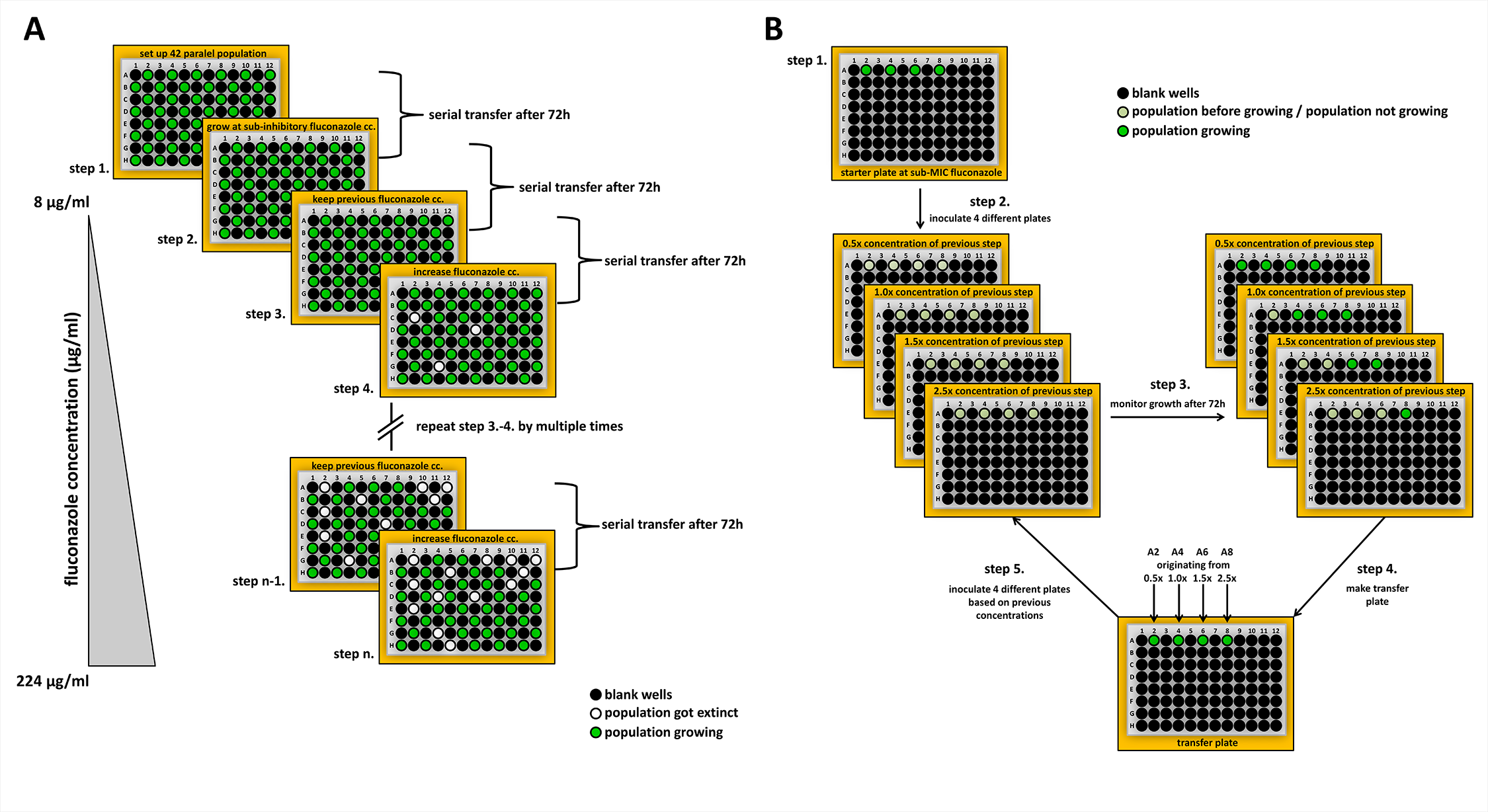

Supplement: S2 Fig — Concept figure of the laboratory evolution protocols. (A) Experiment A measured the extinction rate of the evolving populations as a function of gradually increasing fluconazole dosage. The concept figure shows the basic steps of Experiment A. At the first step, 42–42 independent populations of noPF and PF strains were set up (color-coded as green wells), respectively. The noPF and PF populations were evolved parallel on different 96 well plates. Checker board layout was used on the plate to monitor cross-contamination events. Blank wells are color-coded as black wells. 10% of the populations were serially transferred into fresh medium every 72 hours. Starting at a sub-inhibitory fluconazole concentration (step 2., 8 μg/ml), fluconazole dosage was increased gradually at every second transfer (e.g. at step 4., after applying the same concentration as at step 3.). The applied dosages were as follows: 0, 8, 16, 24, 32, 64, 96, 128, 160, 192 and finally 224 μg/ml. Population extinction (color-coded as white wells) was defined by a cut-off OD600 = 0.15 after 72 hours of cultivation. Evolved strains from the final day of Experiment A (step n.; after reaching resistance towards 224 μg/ml fluconazole) were used for further genomic and functional analyses. (B) Experiment B aimed to maximize fluconazole resistance increment during a fixed time period. The concept figure shows the basic steps of Experiment B. The same medium and culturing conditions were used as in Experiment A, using the same checker board layout (blank wells are color-coded as black). For simplified representation purposes, only the A2, A4, A6 and A8 wells of the microtiter plate are considered here as evolving populations. Growing populations are color-coded as green wells, whereas populations before growth or populations that did not grow are color-coded as faint green. Starting with sub-inhibitory drug concentration (step 1.), each population was allowed to grow for 72 hours. Populations were transferred [file pbio.2000644.s002.tif]

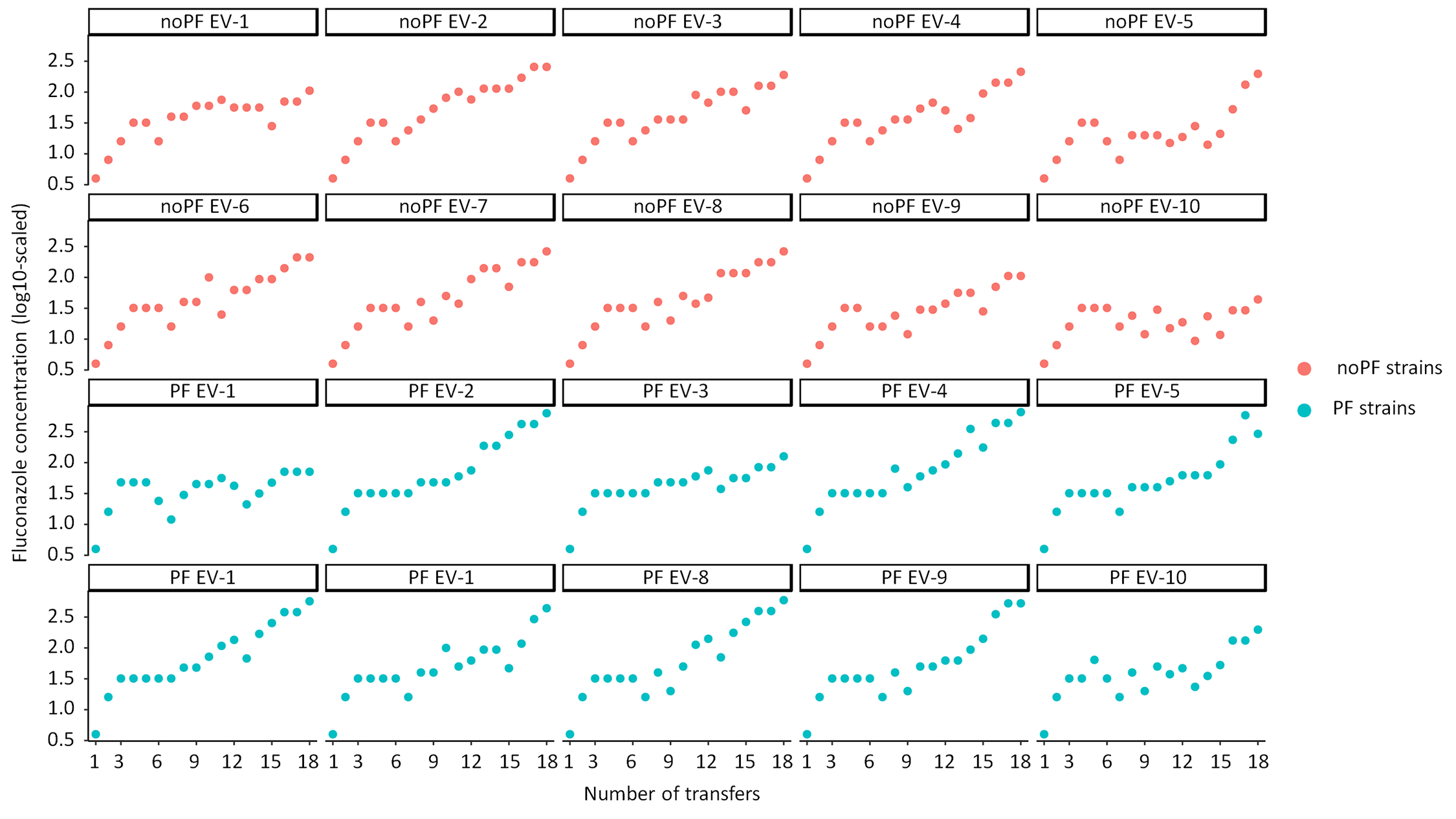

Supplement: S3 Fig — The figure shows the resistance level of ten independent populations of noPF (blue circles) and ten independent populations of PF (red circles) strains, as a function of time (transferring steps). The data underlying S3 Fig can be found in S1 Data. (TIF) [file pbio.2000644.s003.tif]

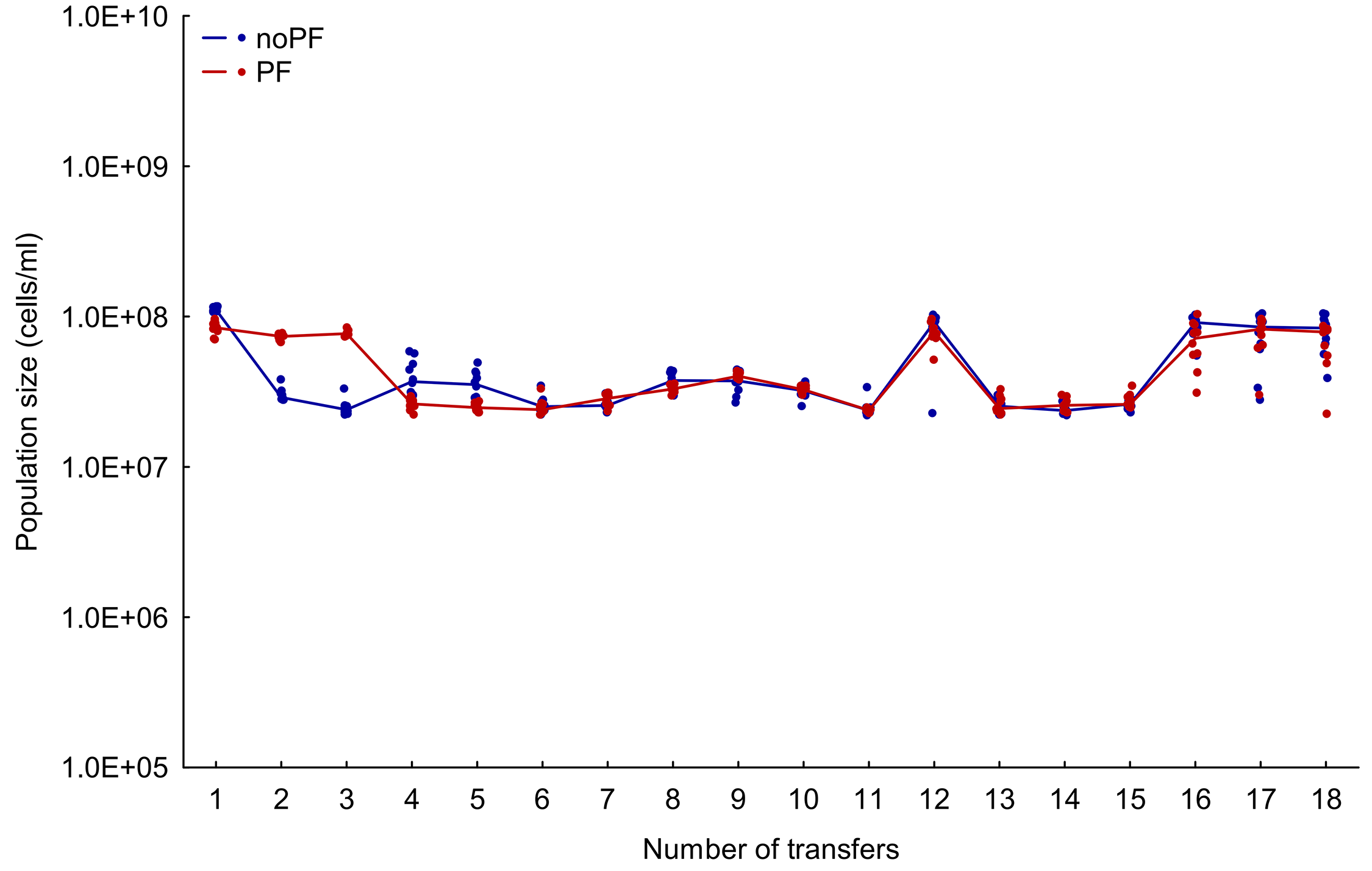

Supplement: S4 Fig — The figure shows the population size (cells/ml) of noPF and PF strains (after 72 hours of growth) as a function of transferring steps. We found no significant difference in the population size between evolving PF and noPF populations (paired t-test, P = N.S.). See Materials and methods for evaluation of population size. The data underlying S4 Fig can be found in S1 Data. (TIF) [file pbio.2000644.s004.tif]

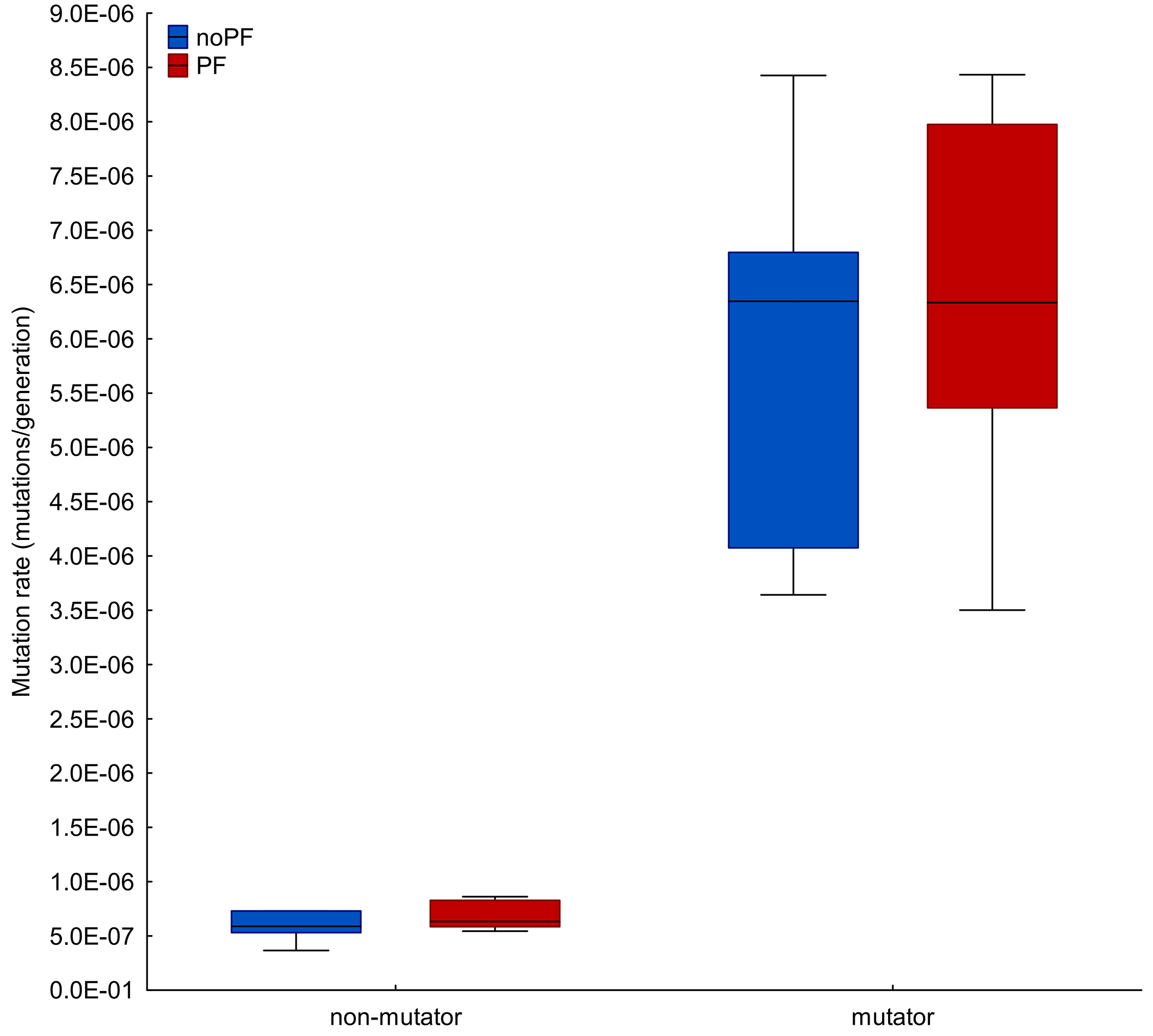

Supplement: S5 Fig — The boxplot shows the mutation rates of non-mutator and mutator noPF (red) and PF strains (blue), respectively. The box shows the upper and lower quartiles, the black line the median (based on six replicates), and the whiskers the non-outlier range. There was no significant difference in the mutation rate between non-mutator PF and non-mutator noPF strains (t-test, P = not significant), and between mutator PF and mutator noPF strains (t-test, P = not significant). This result is consistent with the results of whole genome sequence analysis of the evolved strains: There was no significant difference in the number of mutations between noPF and PF strains (Fig 5A, main text). The mutator strains show an average 10-fold increase in mutation rate relative to their corresponding non-mutator strains. See Materials and methods for evaluation of population size. The data underlying S5 Fig can be found in S1 Data. (TIF) [file pbio.2000644.s005.tif]

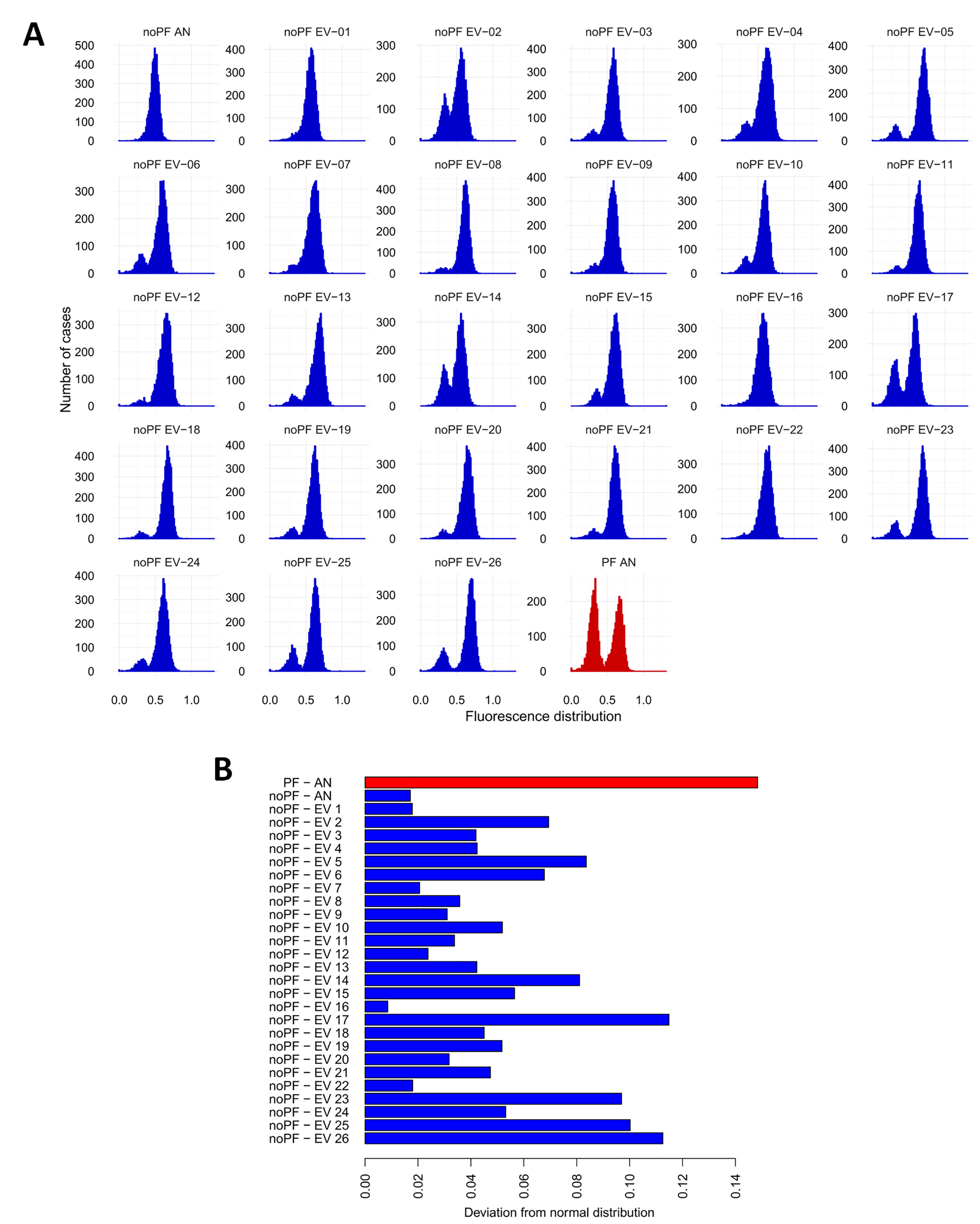

Supplement: S6 Fig — (A) The histograms show the fluorescence distributions of PDR5-GFP in noPF evolved strains (blue, noPF1-26), noPF ancestor (blue, noPF AN) and PF ancestor (red, PF AN). (B) Deviation of the fluorescence distributions from the normal distribution. The barplot shows the deviation (D value) of the fluorescent distributions in PF ancestor (PF AN, red), noPF ancestor (noPF AN, blue) and noPF evolved strains (noPF EV1-26, blue). The larger the D value is, the larger the deviation from the expected normal distribution. To calculate D value, a standard Kolmogorov-Smirnov normality test was performed. The data underlying S6 Fig can be found in S1 Data. (TIF) [file pbio.2000644.s006.tif]

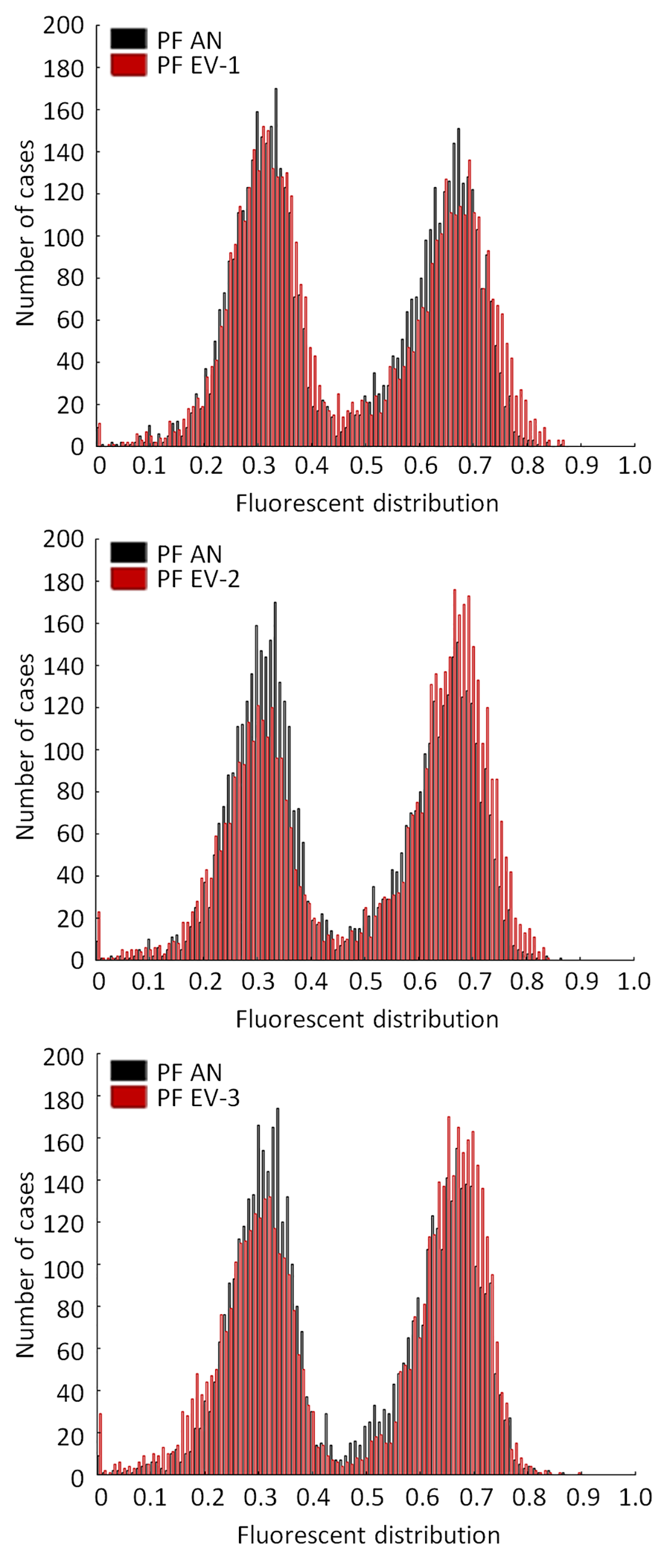

Supplement: S7 Fig — The histograms show the fluorescence distributions of PDR5-GFP in three representative evolved PF strains (red, PF EV-1-3) and PF ancestor (black, PF AN). The data underlying S7 Fig can be found in S1 Data. (TIF) [file pbio.2000644.s007.tif]

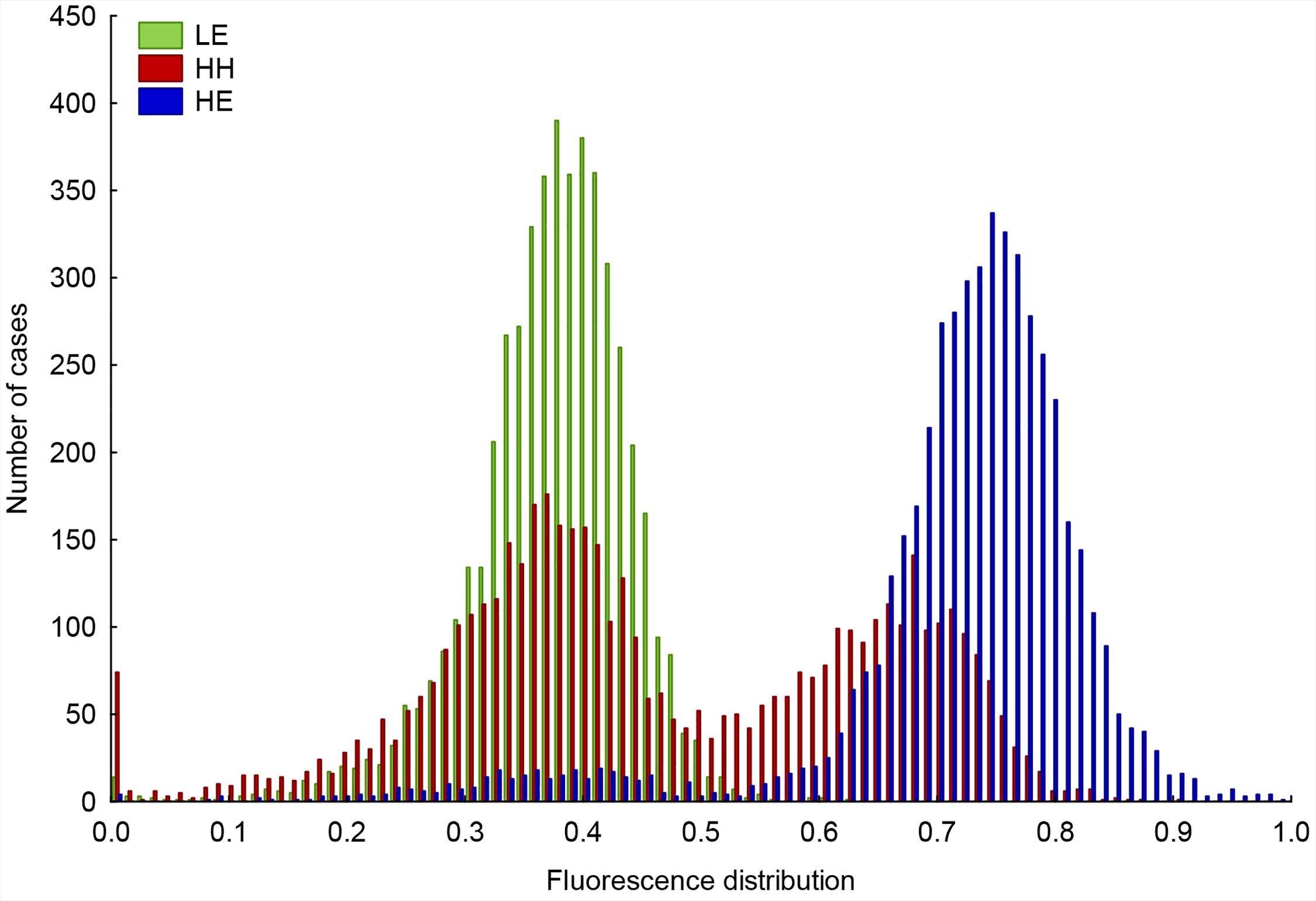

Supplement: S8 Fig — The histograms show the fluorescence distributions of PDR5-GFP for the ancestor PF strain at three different expression states: low expression level (LE, induced by 0.015 μg/ml doxycyclin), high heterogeneity (HH, induced by 0.3 μg/ml doxycyclin) and high expression level (HE, induced by 3 μg/ml doxycyclin). Both the mean of expression and coefficient of variation differed in HE and LE state, compared to the HH state: the mean was higher by 54% for HE (Mann Whitney U-test, P < 0.001) and lower by 20% for LE (Mann Whitney U-test, P < 0.001), while the coefficient of variation was lower by 52% and 57%, respectively (Mann Whitney U-test, P < 0.001). The data underlying S8 Fig can be found in S1 Data. (TIF) [file pbio.2000644.s008.tif]
